# Genetic Insight Into the Insect Resistance in Bread Wheat Exploiting the Untapped Natural Diversity

**DOI:** 10.3389/fgene.2022.828905

**Published:** 2022-02-11

**Authors:** Mian Abdur Rehman Arif, Muhammad Qandeel Waheed, Ulrike Lohwasser, Sajid Shokat, Ahmad M. Alqudah, Christa Volkmar, Andreas Börner

**Affiliations:** ^1^ Wheat Breeding Group, Plant Breeding and Genetics Division, Nuclear Institute for Agriculture and Biology, Faisalabad, Pakistan; ^2^ Leibniz Institute of Plant Genetics and Crop Plant Research, Gatersleben, Germany; ^3^ Department of Agroecology, Aarhus University at Flakkebjerg, Slagelse, Denmark; ^4^ Institute of Agricultural and Nutritional Sciences, Martin-Luther-University Halle-Wittenberg, Halle, Germany

**Keywords:** wheat, OWBM, YWBM, SGM, candidate genes, SGM, frit fly, thrips

## Abstract

Climate change is an undeniable threat to sustainable wheat production in the future as an increased temperature will significantly increase grain loss due to the increased number of generations per season of multivoltine species that are detrimental to plants. Among insects, orange wheat blossom midge (OWBM), yellow wheat blossom midge (YWBM), saddle gall midge (SGM), thrips, and frit fly (FF) are important wheat pests in the European environments, which can be managed by the development of resistant cultivars. This involves the identification, confirmation, and incorporation of insect resistance sources into new high-yielding cultivars. We used two diverse and unrelated wheat [winter wheat (WW) and spring wheat (SW)] panels to associate single-nucleotide polymorphism (SNP) markers with the mentioned pests using the tools of association mapping. All in all, a total of 645 and 123 significant associations were detected in WW and SW, respectively, which were confined to 246 quantitative trait loci. Many candidate genes were identified using the BLAST analysis of the sequences of associated SNPs. Some of them are involved in controlling the physical structures of plants such as stomatal immunity and closure, cuticular wax in leaf blade, whereas others are involved in the production of certain enzymes in response to biotic and abiotic stresses. To our knowledge, this is the first detailed investigation that deals with YWBM, SGM, thrips, and FF resistance genetics using the natural variation in wheat. The reported germplasm is also readily available to breeders across the world that can make rational decisions to breed for the pest resilience of their interest by including the resistant genotypes being reported.

## Introduction

Wheat (*Triticum aestivum* L.) productivity and global food security have become synonymous with each other as wheat is the most important food crop in major parts of the world (Shiferaw et al., 2013; Curtis and Halford, 2014). During the last decade, global wheat production increased from 655 million metric tons (MT) (in 2011–2012) to 772 million MT (in 2020–2021) (https://www.statista.com/statistics/267268/production-of-wheat-worldwide-since-1990/), making a 10.76% increase in 10 years (1% per annum) (Table 1). Because there will be 9 billion people to be fed by 2050, the current wheat yield should be doubled by 2050 (Ray et al., 2013). This is only possible, however, if the yield is increased at the rate of 1.66% instead of 1% per annum. This prompts the demand to use, adopt, and utilize all the available tools and resources to sustain and increase the current wheat production (Hasan et al., 2020) to ensure food security for next generations.

**TABLE 1:** The global increase in wheat yield per year in the last decade.

Year	Global production	% Increase or decrease
2011–2012	697	
2012–2013	655	−6.02
2013–2014	717	9.46
2014–2015	728.3	1.57
2015–2016	735.9	1.04
2016–2017	756.5	2.799
2017–2018	762	0.72
2018–2019	730.9	−4.08
2019–2020	763.93	4.51
2020–2021	772	1.05
Decade		10.76%

Recent reports (Deutsch et al., 2018) have shown that an increased temperature will significantly increase the grain loss in many parts of the world in wheat, maize, and rice. Likewise, another impact of climate change would be an increased number of generations per season of multivoltine species and their accelerated development causing potentially more damage to crops (Crespo-Herrera et al., 2019). Therefore, climate change will likely threaten sustainable wheat production in the future (Crespo-Herrera et al., 2019). In addition, wheat production has been threatened by unexpected abiotic and biotic stresses due to abrupt environmental changes or the movement of pathogens (Bakala et al., 2021).

The agents responsible for biotic stresses include fungi, viruses, insects, nematodes, arachnids, and weeds, directly affecting plant growth and development by depriving them of nutrition resulting into reduced plant vigor coupled with low yield (Bakala et al., 2021). Among insects, orange wheat blossom midge (OWMB), *Sitodoplosis mosellana* (Géhin), is recognized as one of economically important insects (Zhang et al., 2020). Losses due to this pest can go up to 30% in yield responsible for an economic loss of 30 million dollars (Kamran et al., 2013). The 1950s’ and 1980s’ outbreaks of OWBM in China witnessed 50% yield reduction in wheat production (Duan et al., 2013). The emergence of adult OWBM is synchronized with wheat heading where they lay eggs on the spikelets possibly due to certain wheat odor components (Birkett et al., 2004). On the other hand, young kernels are eaten by larvae; the result being reduced yield and quality (Olfert et al., 1985; Lamb et al., 2000). After harvest, these larvae overwinter in the soil, migrate to the soil surface in the spring, re-enter the soil to pupate, and then emerge from the soil as adults to infect the next crop cycle (Blake et al., 2011).

Another insect is the yellow wheat blossom midge (YWBM), *Contrinia tritici* (Kirby) (El-Wakeil et al., 2013), that is a univoltine species stayed overwinter in the soil, which pupate while arriving at the soil surface under ambient conditions culminating into adults in spring and starting mating. The mated females find young spikes to lay eggs. Like OWBM, YWBM larvae also feed on young kernels. In early July, larvae slip from spikes/stems at the advent of rain and burrow into the soil, where they enter diapause (Barnes, 1956).

An important biotic impediment in wheat productivity in Europe, in addition to OWBM and YWBM, is the saddle gall midge (SGM), *Haplodiplosis marginata* (von Roser, 1840). Contrary to OWBM and YWBM, the larvae of SGM feed on stems (Harris et al., 2001). SGM is the least studied of the three midges and has caused severe damage in cereals in recent years in Western Europe (Censier et al., 2012; Dewar, 2012). Its life cycle is also like the OWBM and YWBM. Its emergence is favored by warm and humid weather (Golightly, 1979). After emergence, females mate and lay eggs on both sides of young leaves (Golightly, 1979), which mature into larvae under conducive circumstances. These larvae crawl under the leaves and feed on the stem (Barnes, 1956). Each larva makes a small longitudinal depression giving rise to galls. The stem tissue at end of the gall forms two bulges (Balachowsky and Mesnil, 1935). Nutrient flow in a plant stem is intercepted as a result (Golightly, 1979).

In addition to midges, wheat is also attacked by thrips. The main species include *Limothrips denticornis*, *L. cerealium*, *Haplothrips tritici*, and *H. aculeatus* (El-Wakeil et al., 2010). Both winter wheat (WW) and spring wheat (SW) are affected (Andjus, 1996; Moritz, 2006). The first generation is completed in winter cereals followed by a second generation on SW (Köppä, 1970). Distortion, degeneration, and grain abortion is the result after thrips feeding on juvenile ears. Major effects include partial or complete discoloration of the ears (white ear effect), drying of the flag leaf, partial ear fertilization, and incomplete grain filling (15%–31% grain weight loss) (Larsson, 2005).

Frit fly (FF) (*Oscinella frit*) is another neglected biotic stress insect as it is not identified in Western European research. Hence, the losses due to FF are seen in the Czech Republic, Germany, Hungary, Romania, and Sweden (Ricroch, 2017). FF is a stem borer capable of causing considerable loss to spring cereals (El-Wakeil and Volkmar, 2011) and to winter cereals when sown early. The larva of FF overwinters within the stems of cereal plants (Lindblad, 1999) and pupates in spring. Females lay eggs on SW seedlings behind the coleoptile (Jonasson, 1977). Main shoot destruction is witnessed when the larva penetrates the plant causing yield losses (Lindblad and Sigvald, 1999).

It is evident from the above introduction that these pests are to be managed to improve farmers’ income and profitability. They are, however, hard to manage due to their small sizes, ability to use alternate hosts, and diapausing in the soil for prolonged periods (Barnes, 1956; Capinera, 2008; Censier et al., 2015). The main control strategies are based on insecticide treatments (Chavalle et al., 2015; Censier et al., 2016; Chavalle et al., 2018). However, because their occurrence is extremely environment-dependent, it is difficult to time insecticide applications and monitor populations to stop the outbreaks (Chavalle et al., 2015). Other strategies are using biopesticides such as insect pathogens (El-Wakeil et al., 2013; Shrestha and Reddy, 2019).

An alternate strategy to cope with the abovementioned insects is the development of resistant cultivars, which involves the identification, confirmation, and incorporation of insect resistance sources into new high-yielding cultivars. Limited success, however, has been achieved in the case of only OWBM (DePauw et al., 2009; Vera et al., 2013; https://ahdb.org.uk/) after the identification of the first antibiosis gene *Sm1* mapped on chromosome 2BS from American wheat variety “Augusta” (McKenzie et al., 2002; Berzonsky et al., 2003). This gene has been incorporated in 30 bread and durum SW varieties in Europe and North America (Lamb et al., 2001; Gaafar et al., 2011; Blake et al., 2014). Two routes are available to determine the number and location of candidate genes underlying natural variation in any quantitatively inherited trait: *via* biparental linkage mapping and *via* phenotype–genotype association analysis (Zhu et al., 2008; Arif et al., 2017).

In this investigation, we aimed to get an insight into the genetic architecture of OWBM, YWBM, SGM, thrips, and FF in two different sets of wheat (WW and SW) panels using the latter. The primary aim was to assess the natural phenotypic variation against naturally existing populations of the abovementioned insects in various parts of central Germany. Another objective is to associate molecular markers with the differential phenotypic response to map loci underlying the resistance to these pests. Here, we reported that many promising novel quantitative trait loci (QTL) control the resistance of multi-insects, which can be implemented in wheat breeding for grain yield improvement. Finally, using the sequences of the associated markers, the genes located at the site of detected QTLs were sought.

## Materials and Methods

### Plant Materials

The plant materials used to achieve the objectives mentioned in introduction part consisted of two wheat panels known as WW and SW panels. Both panels were grown in plots (2 × 1.5 m) in one replication but on different experimental sites [Gatersleben at Institute for Plant Genetics and Crop Plant Research (IPK-Gatersleben) and Quedlinburg at Julius Kühn Institute (JKI, Quedlinburg), state Saxony Anhalt; Rosenthal, state Lower Saxony; Oberpleichfeld state Bavaria)] and in different years between 2011 and 2016. Details are given in Supplementary Table S8.

The WW panel was composed of 96 WW accessions assembled at the Institute of Field and Vegetable Crops, Novi Sad, Serbia; accessions were selected on the basis of their phenotypic diversity with respect to a group of key agronomic traits, and their provenance is spread over 21 countries (Alqudah et al., 2020a). Initially, the panel was genotyped with 525 mapped and 315 unmapped DArT markers (Arif et al., 2012a), which resulted in the pioneer studies related to seed longevity (Arif et al., 2012a), dormancy, and pre-harvest sprouting (PHS) (Arif et al., 2012b). With the arrival of single-nucleotide polymorphisms (SNPs), this panel was genotyped with 15K Infinium SNP array, resulting in 11,139 SNPs that were mapped to all 21 linkage groups of bread wheat (Alqudah et al., 2020a). Recently, a re-analysis of the data of Rehman-Arif et al. (Arif et al., 2012a) with the new SNP data in this panel has revealed interesting loci of seed longevity in wheat (Arif and Börner, 2020).

The SW panel was composed of 111 accessions assembled from a very large collection of wheat resources at the IPK-Gatersleben on the basis of the differential behavior of seed survival. Initially, a set of 183 hexaploid wheat (129 spring type and 54 winter type) accessions (Arif et al., 2017) was selected from the collection maintained at the IPK Genebank and last multiplied in 1974, constituting the oldest seed lots available in the storage. In the beginning, it was mapped with the 2,134 polymorphic DArT markers covering a genetic distance of 2,875 cM (Arif et al., 2017). Later on, the panel was reduced to 111 on the basis of on-field behavior, provenance, and growth habit. To attain better marker coverage, these accessions were re-genotyped with a 15K Infinium SNP array. The result was the mapping of 9,804 high-quality SNPs covering a distance of 3,624.71 cM (2.70 SNPs/cM) on all the linkage groups of bread wheat (Arif and Börner, 2020). This 15K SNP Infinium SNP array is an upgraded, refined, and narrowed version of the 90K iSELECT SNPchip (Wang et al., 2014). The panel has been successfully used to elucidate the loci linked with anther extrusion (Muqaddasi et al., 2017) and more recently with seed longevity (Arif and Börner, 2020).

### Phenotyping

The germplasm was screened for resistance to natural population of the following five insects, *viz*., *Sitodiplosis mosellana* (OWBM), *Contarinia tritici* (YWBM), *Haplodiplosis marginata* (SGM), thrips, and *Oscinella frit* (FF).

For OWBM and YWBM, the numbers of larvae in spikes (LS) and adults and larvae in white traps/shells (AWS and LWS, correspondingly) were counted as a measure of resistance. White water traps/shells were used to sample migrating (from ears to soil) midges (adults and larvae). The traps consisted of white plastic dishes: 12.5 cm diameter and 6.5 cm deep. One trap was placed in each plot on the ground among wheat plants from early June until the end of July. Traps were partly filled with water plus few drops of detergent. Caught adults and larvae were counted once per week using a magnifying glass (Gaafar et al., 2011).

On the other hand, the numbers of larvae per ear were assessed by collecting five to eight ears randomly per plot at approximately Zadoks stage 73 (Zadoks et al., 1974). Samples were put into a bag that was tightly sealed and stored at −20°C. After finishing the growing season, the ears were dissected under a binocular (SMZ645, Nikon), and the numbers of larvae were counted (Gaafar et al., 2011).

This methodology has been successfully adopted to identify wheat varieties most resistant to wheat ear insect pests in Central Germany by Gaafar et al. (2011). They used two methods to evaluate the degree of insect infestations in ears of different wheat varieties. One was inspection of wheat ears to count the number of spikelets and infested kernels and to identify the insect pests present. The second was the use of white water traps/shells to collect mature larvae of midges under consideration as an indicator of potential crop risk.

For SGM, ∼20 tillers per plot were randomly selected at approximately Zadoks stage 55 (Zadoks et al., 1974). Within 14 days after cutting, the number of saddles per tiller and the total number of saddles caused by the insects were counted.

The numbers of larvae and adult individuals per ear in case of thrips were assessed by collecting five to eight ears per plot. The time of collection and the handling of the ears was the same as described for OWBM and YWBM. Finally, the infestation with *Oscinella frit* was examined in autumn (WW panel) and/or in spring (WW and SW panels). The number of damaged seedlings in two middle rows of the plots and on a length of 2 m was counted.

### Statistical and Genetic Analyses

All the basic phenotypic analyses including ANOVA and broad sense heritability (*h*_*2*_) were conducted in RStudio version 1.3.1093. Histograms were constructed using “ggplot2” package and pairwise comparisons were carried out using “ggpubr” package. Pearson’s correlation coefficient among the phenotypic traits were caclulated at *p* ≤ 0.05 for correlation networks that were visualized using “qgraph” package (Epskamp et al., 2012) for the significant correlations.

Details about genetic analyses and GWAS are provided in (Arif and Börner, 2020; Alqudah et al., 2020b). Briefly, genotypic data of both WW and SW were subjected to population structure analysis prior to association mapping using *STRUCTURE v.2.3.4* (Pritchard et al., 2000) applying the admixture model, a burn-in of 100,000 iterations and 100,000 MCMC duration to test for a K-value in the range 1–15. The results were subjected to Structure Harvester (Earl and VonHoldt, 2012) for better visualization, which is available elsewhere (Arif and Börner, 2020). According to Arif et al. (Arif and Börner, 2020), there were three and four subgroups in WW and SW panels, correspondingly. We carried out the association analyses harnessing the program *TASSEL 5.2.43* (Bradbury et al., 2007), employing mixed linear model (Yu et al., 2006) considering the population structure (calculated from *STRUCTURE v.2.3.4*) and kinship (calculated from *TASSEL 5.2.43*). Because the information about genetic analyses on insect resistance is very scarce, we considered all the SNPs significant that gave a *p*-value of 0.001 (−log10 value of 3) for any trait. Highly significant *p*-values were calculated by taking the reciprocal of the number of markers for each set. Therefore, *p*-values of 8.97 × 10^−5^ and 1.019 × 10^−-4^ were considered for highly significant association in WW and SW, respectively. Results from *TASSEL* were visualized using the “CM plot” package in R. For QTLs visualization, the “circlize” package (Gu et al., 2014) was utilized. QTLs were named following the rules set out in the Catalog of Gene Symbols (McIntosh et al., 2008) and according to our previous reports (Arif and Börner, 2019; Arif et al., 2021). The markers were mapped on the basis of their physical position in IWGSC RefSeq v1.1 (http://www.wheatgenome.org/, IWGSC RefSeq v1.1). The highly significant associated SNPs were used to identify the high-confidence putative candidate genes on the basis of their physical positions.

The sequences of flanking SNPs within the linkage disequilibrium (LD) of associated SNP with all the insects (i.e., multi-insect traits SNP) were obtained from the Wheat 15 and 90K SNP array database (Wang et al., 2014). These sequences were used as a query in NCBI BLASTX (https://blast.ncbi.nlm.nih.gov/Blast.cgi?PROGRAM=blastx&PAGE_TYPE=BlastSearch&LINK_LOC=blasthome) research tool database for functional gene annotations. The topmost hits with the smallest E-value and high percentage of query coverage were reported as potential candidate genes.

## Results

### Descriptive Natural Phenotypic Variation

#### Orange Wheat Blossom Midge

Among all insects, OWMB was the most intensively investigated because of its frequent natural occurrence as compared to other pests. All in all, it was scored in at least seven [for larvae in white shells (WSL)] to 10 environments [for larvae on spikes/ears (SL) and adults in the white shell (WSA)] in WW. The mean of SL, among all 10 environments, was highest in G13 (13.30 ± 1.25) followed by Q13 (4.50 ± 0.29) (Supplementary Figure S1, Table 2). Likewise, mean SL was comparable between G11 (1.73 ± 0.16) and Q16 (1.79 ± 0.33) and between G12 (0.94 ± 0.14) and R12 (0.82 ± 0.10). Mean SL in R13 was 1.47 ± 0.13, whereas it was 0.55 ± 0.12 in Q15. Finally, the lowest SL score was observed in G15 (0.12 ± 0.02) followed by Q12 (0.39 ± 0.10). From 44 pairwise comparisons, non-significant differences were detected in only six pairs (G11 and R13, G12 and R12, G15 and Q15, G15 and R13, Q12 and Q16, Q12 and R13, and Q15 and R13) (Supplementary Figure S2A). WSL was highest in G14 (35.76 ± 3.20) followed by Q13 (25.37 ± 1.57) followed by G11 (23.96 ± 2.15) and lowest in Q12 and G15 (0.20 ± 0.05). WSL in G12 and Q15 was 3.61 ± 0.36 and 7.88 ± 0.78, respectively. Moreover, significant differences were also prevalent among all combinations except between G11 and G15, G14 and Q13, and G15 and Q12 (Supplementary Figure S2B). As per WSA was concerned, it was highest in Q13 (46.03 ± 1.75), followed by R12 (35.76 ± 3.20). The scores of WSA were comparable between G13 (11.69 ± 1.17) and Q15 (13.69 ± 0.60) and between G11 (7.73 ± 0.61) and G15 (8.67 ± 0.39). Likewise, it was also comparable between Q12 (0.63 ± 0.08) and G14 (0.82 ± 0.10). Finally, the mean WSA was 4.87 ± 0.29 and 3.18 ± 0.20 in G12 and R13, correspondingly. In addition, all 44 combinations of 10 environments were significantly different except between G11 and R13, G12 and R13, and G13 and G15 (Supplementary Figure S2C).

**TABLE 2:** Descriptive statistics of various traits of investigated insects in winter wheat (WW) panel. For details, see materials and methods.

Trait	Range	Mean ± SE	Var	Kurtosis	Skewness	Confidence level (95.0%)	Broad sense heritability (combined)
O_SL_G11	0–7.83	1.73 ± 0.16	2.75	2.18	1.46	0.33	0.23
O_SL_G12	0–6.625	0.94 ± 0.14	2.01	5.4	2.33	0.28	
O_SL_G13	0–65.75	13.30 ± 1.25	152.02	2.87	1.5	2.49	
O_SL_G15	0–1.5	0.12 ± 0.02	0.05	12.49	3.19	0.04	
O_SL_R12	0–1.5	0.16 ± 0.02	0.08	6.97	2.47	0.05	
O_SL_R13	0–7	1.47 ± 0.13	1.64	2.66	1.22	0.26	
O_SL_Q12	0–8.4	0.39 ± 0.10	1.01	45.44	6.01	0.2	
O_SL_Q13	0–12	4.50 ± 0.29	8.23	−0.3	0.46	0.59	
O_SL_Q15	0–6	0.55 ± 0.12	1.53	8.83	2.91	0.25	
O_SL_Q16	0–17	1.79 ± 0.33	10.48	8.28	2.69	0.65	
O_WSL_G11	1–113	23.96 ± 2.15	446.97	2.6	1.45	4.28	0.41
O_WSL_G12	0–21	3.61 ± 0.36	12.21	5.97	1.82	0.71	
O_WSL_G15	0–4	0.20 ± 0.06	0.35	18.26	3.84	0.12	
O_WSL_Q12	0–3	0.20 ± 0.05	0.29	9.63	3	0.11	
O_WSL_Q13	0–64	25.37 ± 1.57	231.32	−0.27	0.46	3.13	
O_WSL_Q15	0–34	7.88 ± 0.78	59.89	1.02	1.28	1.56	
O_WSL_G14	0–192	35.76 ± 3.20	987.8	5.41	1.79	6.36	
O_WSA_G11	0–27	7.73 ± 0.61	36.08	0.68	0.95	1.21	0.19
O_WSA_G12	0–14	4.87 ± 0.29	7.98	−0.02	0.52	0.57	
O_WSA_G13	0–70	11.69 ± 1.17	131.32	6.86	2.13	2.33	
O_WSA_G14	0–4	0.82 ± 0.10	1.09	0.7	1.2	0.21	
O_WSA_G15	2–27	8.67 ± 0.39	15.08	4.06	1.34	0.78	
O_WSA_Q12	0–4	0.63 ± 0.08	0.71	2.24	1.45	0.17	
O_WSA_Q13	10–91	46.03 ± 1.75	285.01	−0.41	0.42	3.47	
O_WSA_Q15	1–35	13.69 ± 0.60	34.76	1.17	0.74	1.19	
O_WSA_R12	0–22	6.08 ± 0.50	23.88	1.8	1.41	1.01	
O_WSA_R13	0–9	3.18 ± 0.20	4.11	−0.01	0.47	0.41	
Y_SL_G11	0–12.83	1.03 ± 0.19	3.73	15.62	3.38	0.39	0.35
Y_SL_G12	0–8	1.03 ± 0.18	3.1	4.33	2.11	0.35	
Y_SL_G13	0–31.12	6.36 ± 0.75	54.44	1.54	1.48	1.49	
Y_SL_Q12	0–3.85	0.17 ± 0.07	0.45	21.42	4.62	0.14	
Y_SL_Q13	0–5.2	0.47 ± 0.09	0.84	7.63	2.52	0.18	
Y_SL_Q16	0–10	1.60 ± 0.25	5.96	2.6	1.83	0.49	
Y_WSL_G11	0–46	10.55 ± 1.05	106.35	2.69	1.71	2.08	0.31
Y_WSL_G12	0–33	7.87 ± 0.73	50.65	1.05	1.16	1.45	
Y_WSL_G13	0–8	1.48 ± 0.16	2.5	3.23	1.63	0.32	
Y_WSL_G14	0–63	7.36 ± 1.12	120.9	109.3	3.09	2.22	
Y_WSL_G15	0–51	8.44 ± 0.98	93.53	6.12	2.27	1.95	
Y_WSL_Q12	0–29	4.54 ± 0.50	23.19	7.03	2.23	0.99	
Y_WSL_Q13	0–42	7.04 ± 0.89	73.82	3.11	1.73	1.76	
Y_WSL_Q15	0–11	1.33 ± 0.22	4.71	7.05	2.39	0.43	
Y_WSA_G12	0–35	3.89 ± 0.54	27.48	17.84	3.68	1.07	0.69
Y_WSA_G13	0–284	60.74 ± 5.68	3072.4	2.92	1.57	11.29	
Y_WSA_G14	0–6	0.88 ± 0.11	1.23	3.94	1.67	0.22	
Y_WSA_G15	0–4	0.54 ± 0.07	0.54	4.75	1.77	0.14	
Y_WSA_Q12	Feb-66	28.96 ± 1.17	125.98	1	0.62	2.32	
Y_WSA_Q13	0–13	4.69 ± 0.25	6.03	0.41	0.53	0.5	
Y_WSA_Q15	0–2	0.29 ± 0.05	0.31	2.32	1.8	0.11	
H_O12	0–178	34.57 ± 4.48	1671.37	2.64	1.75	8.92	0.26
H_O13	0–149	39.6 ± 4.41	1854.43	−0.3	0.94	8.77	
H_R13	0–15	1.34 ± 0.33	11.04	7.99	2.89	0.67	
T_G11	0–27	8.88 ± 0.51	25.79	1.31	1	1.02	0.32
T_G12	2.25–28.62	10.10 ± 0.57	32.27	0.7	0.98	1.15	
T_G13	1.87–35.12	13.13 ± 0.68	45.26	1.16	1.11	1.36	
T_Q12	0–9.83	1.87 ± 0.18	2.98	5.18	1.94	0.35	
T_Q13	0–14.125	3.15 ± 0.26	6.88	3.01	1.5	0.53	
T_Q15	10–405	86.72 ± 7.19	4963.04	5.06	1.96	14.27	
T_Q16	8–249	81.90 ± 4.46	1915.43	1.15	0.86	8.86	
T_R12	0–22.625	5.30 ± 0.47	21.71	3.1	1.62	0.94	
T_R13	0–8.89	4.42 ± 0.18	3.22	−0.41	0.26	0.36	
F_GA13	0–3.53	0.90 ± 0.09	0.874	0.38	1.04	0.19	0.25
F_GA14	0–4.78	1.43 ± 0.08	0.75	2.24	1.11	0.17	
F_QA13	0–10.60	3.98 ± 0.23	5.47	0.02	0.6	0.47	
F_GS15	0–5.31	1.98 ± 0.10	1.01	1.3	0.78	0.2	0.25
F_QS14	2.77–87.67	28.99 ± 1.47	208.3	2.44	1.11	2.92	

In SW, scores were available from five environments (G12, G13, G14, G15, and Q15). The SL score was highest in G12 (63.83 ± 4.00), followed by G13 (12.59 ± 0.68) in SW (Supplementary Figure S3, Table 3). On the other hand, G15 exhibited the lowest SL score (1.69 ± 0.15), whereas Q15 exhibited a score of 3.06 ± 0.23. To add to it, all scores were significantly different from each other (Supplementary Figure S4A). WSL was scored only in G13 and Q14 with corresponding values of 8.69 ± 1.02 and 11.37 ± 0.97. They were also significantly different from each other (Supplementary Figure S4B). On the other hand, WSA was scored in all five environments where the highest value was observed in G12 (13.63 ± 0.52), followed by G13 (12.72 ± 0.55). WSL in Q14 and Q15 was comparable (2.26 ± 0.19 and 1.91 ± 0.17, respectively), whereas it was 4.72 ± 0.129 in G15. Furthermore, significant differences prevailed among all comparisons except between G12 and G13 and between Q14 and Q15 (Supplementary Figure S4C). ANOVA results also indicated significant differences in all traits of OWBM in both WW (Supplementary Table S1) and SW panels (Supplementary Table S2).

**TABLE 3:** Descriptive statistics of various traits of investigated insects in spring wheat (SW) panel. For details, see materials and methods.

Trait	Range	Mean ± SE	Var	Kurtosis	Skewness	Confidence level (95.0%)	Broad sense heritability (combined)
O_SL_G12	3–192	63.83 ± 4.00	1781.5	0.92	1.01	7.93	0.3
O_SL_G13	1.6–41.2	12.59 ± 0.68	52.42	1.15	0.89	1.36	
O_SL_G15	0–8.4	1.69 ± 0.15	2.58	3.65	1.64	0.3	
O_SL_Q15	0–11	3.06 ± 0.23	6.33	1.3	1.1	0.47	
O_WSL_G13	0–59	8.69 ± 1.02	116.1	4.72	2.03	2.02	0.42
O_WSL_Q14	0–59	11.37 ± 0.97	106.18	3.72	1.59	1.93	
O_WSA_G12	Apr-31	13.63 ± 0.52	30.63	0.34	0.62	1.04	0.25
O_WSA_G13	Mar-45	12.72 ± 0.55	33.94	8.07	2	1.09	
O_WSA_G15	0–16	4.72 ± 0.29	9.52	1.95	1.07	0.58	
O_WSA_Q14	0–10	2.26 ± 0.19	4.32	1.75	1.23	0.39	
O_WSA_Q15	0–10	1.91 ± 0.17	3.27	5.19	1.89	0.34	
Y_SL_G12	7–265	74.60 ± 4.54	2290.15	1.35	0.94	9	0.22
Y_SL_G13	0–38.6	9.15 ± 0.62	42.58	3.19	1.32	1.23	
Y_SL_G14	0–17.37	3.02 ± 0.33	12.36	3.23	1.73	0.66	
Y_SL_G15	0–21	1.41 ± 0.31	10.75	15.43	3.65	0.61	
Y_SL_Q15	0–10	0.43 ± 0.14	2.19	25.66	1.77	0.27	
Y_WSL_G13	0–100	13.51 ± 1.50	250.41	8.63	2.5	2.97	0.21
Y_WSL_G14	0–223	31.36 ± 3.32	1226.39	9.74	2.7	6.58	
Y_WSA_G13	0–6	1.60 ± 0.12	1.85	0.85	0.88	0.25	0.48
Y_WSA_G14	0–10	1.65 ± 0.17	3.48	4.8	1.86	0.35	
Y_WSA_G15	0–8	1.11 ± 0.14	2.44	4.63	2	0.29	
Y_WSA_Q15	0–5	0.82 ± 0.10	1.17	2.67	1.56	0.2	
T_G13	2–35.25	10.95 ± 0.55	34.75	2.67	1.37	1.1	0.18
T_G15	0–26.2	9.49 ± 0.48	26.48	0.82	0.93	0.96	
T_Q15	108–1095	461.48 ± 19.57	42521.27	0.3	0.71	38.78	
F_G13	2.27–43.61	13.04 ± 0.76	65.21	2.58	1.5	1.51	0.14
F_G14	6.15–67.74	24.90 ± 0.16	151.23	1.5	1.11	2.31	
F_G15	1.94–25.49	10.02 ± 0.44	21.86	1.06	0.98	0.87	

### Yellow Wheat Blossom Midge

In the WW panel, among the five environments in which the SL (G11, G12, G13, Q12, Q13, and Q16) was measured, the highest incidence was observed in G13 with a mean value (± standard error) of 6.36 ± 0.75, whereas the lowest incidence was observed in Q12 with a mean value of 0.17 ± 0.07 (Supplementary Figure S5, Table 2). Mean SL in G11 and G12 was similar (1.03 ± 0.19), whereas mean SL in Q13 and Q16 was 0.47 ± 0.09 and 1.60 ± 0.25, respectively. Pairwise comparisons indicated that differences for SL between G11 and G12, G11 and Q13, G11 and Q16, G12 and Q13, and G12 and Q16 were non-significant. All other combinations were significantly different from each other (Supplementary Figure S6A). For WSL, among the eight environments, the highest and lowest scores were observed in G11 (10.55 ± 1.05) and Q15 (0.29 ± 0.05), respectively, whereas, in G12, Q13, and G14, scores were quite similar (7.87 ± 0.73, 7.04 ± 0.89, and 7.36 ± 1.12, respectively). Mean WSL in G13, Q12, and G15 were 1.48 ± 0.16, 4.54 ± 0.50, and 8.44 ± 0.98, correspondingly. Moreover, WSL was significantly different among all pairs except between G11 and Q13, G14 and Q13, and G15 and Q13 (Supplementary Figure S6B). WSA was highest in Q13 (60.75 ± 5.78) followed by Q12 (28.96 ± 1.17) followed by Q13 (4.69 ± 0.25). Mean WSA in G12 was 3.89 ± 0.54. On the other hand, the lowest WSA was observed in Q15 (0.29 ± 0.05) followed by G15 (0.54 ± 0.07) followed by G11 (0.64 ± 0.12) and mean WSA was 3.89 ± 0.54 in G12. WSA was also significantly different among all environments (Supplementary Figure S6C) except between G11 and Q13 and between G11 and Q15.

In SW, traits were scored in five environments (G12, G13, G14, G15, and Q15). For SL, the highest incidence was in G12 where the mean SL was 74.60 ± 4.54 and the lowest incidence was in Q15 with a mean value of 0.43 ± 0.14 (Supplementary Figure S7, Table 3). Mean SL was 9.15 ± 0.62, 3.02 ± 0.33, and 1.41 ± 0.31 in G13, G14, and G15, respectively. In addition, all SL scores were significantly different from each other (Supplementary Figure S8A). WSA was highest in G14 (1.65 ± 0.17) and lowest in Q15 (0.82 ± 0.10), whereas 1.60 ± 0.12 and 1.11 ± 0.14 were the scores in G13 and G15, correspondingly. In pairwise comparisons, except G13, G14, G15, and Q15, all pairs were significantly different (Supplementary Figure S8B). Between G13 and G14, WSL was higher in G14 (31.36 ± 3.32) and lower in G13 (13.51 ± 1.50), which were also significantly different from each other (Supplementary Figure S8C). Significant differences were prevalent across the years in all traits of YWBM in both WW (Supplementary Table S1) and SW panels (Supplementary Table S2).

### Saddle Gall Midge

SGM was recorded in only WW in three environments at Rosenthal and Oberpleichfeld. The highest incidence of SGM was observed in O13 (39.6 ± 4.48) followed by O12 (34.57 ± 4.48) (Supplementary Figure S9), although there was no significant difference between the two (Supplementary Figure S10A). The incidence in R13 was quite low (1.34 ± 0.33), which was also significantly lower than the other two.

### Thrips

Because only one parameter (number of thrips) was measured related to thrips across nine environments, the data were grouped according to the location in both WW and SW. In WW, in Gatersleben, the thrips mean values were 8.88 ± 0.51, 10.10 ± 0.57, and 13.13 ± 0.68 in G11, G12, and G13, correspondingly (Supplementary Figure S11, Table 2). Among the four environments at Quedlinburg, the highest score was observed in Q15 (86.72 ± 7.19) and Q16 (81.90 ± 4.46). On the other hand, mean scores in Q11 and Q12 were 1.87 ± 0.18 and 3.15 ± 0.26, respectively. At Rosenthal, the mean score was higher in R12 (5.30 ± 0.47) than in R13 (4.42 ± 0.18). Thrips scores were significantly different between each other except between G11 and G12, Q15 and Q16, and R12 and R13 (Supplementary Figure S10B).

Among the three environments in SW, the highest score for thrips that was recorded in Q15 was 461.48 ± 19.57 (Supplementary Figure S12A, Table 2). On the other hand, G13 and G15 scores were quite comparable (10.95 ± 0.55 and 9.49 ± 0.48, respectively) with no significant difference between them (Supplementary Figure S13A). The other comparisons were significantly different.

### Frit Fly

FF was scored in two different seasons (S and A) in WW. Among the three environments in the autumn season, the damage was highest in Q13_A (3.98 ± 0.08) and lowest in G14_A (1.43 ± 0.08), whereas the damage was 0.90 ± 0.09 in G13_A (Supplementary Figures S14A,B). Meanwhile, all scores were significantly different from each other (Supplementary Figure S15A). In the spring season, between Q14_S and G15_S, the damage was higher in the former (28.99 ± 1.47) than the latter (1.98 ± 0.10) with a significant difference between the two (Supplementary Figure S15B).

FF damage in SW was also significantly different among the three environments (Supplementary Figure S12B) where the highest damage was observed in G14 (24.90 ± 0.16) and lowest in G15 (10.02 ± 0.44). The damage in G13 was 13.04 ± 0.76 (Supplementary Figure S13B). Thrips and FF scores were also significantly different according to ANOVA results in both WW and SW (Supplementary Tables S1, S2).

### Correlations

**FIGURE 1:**
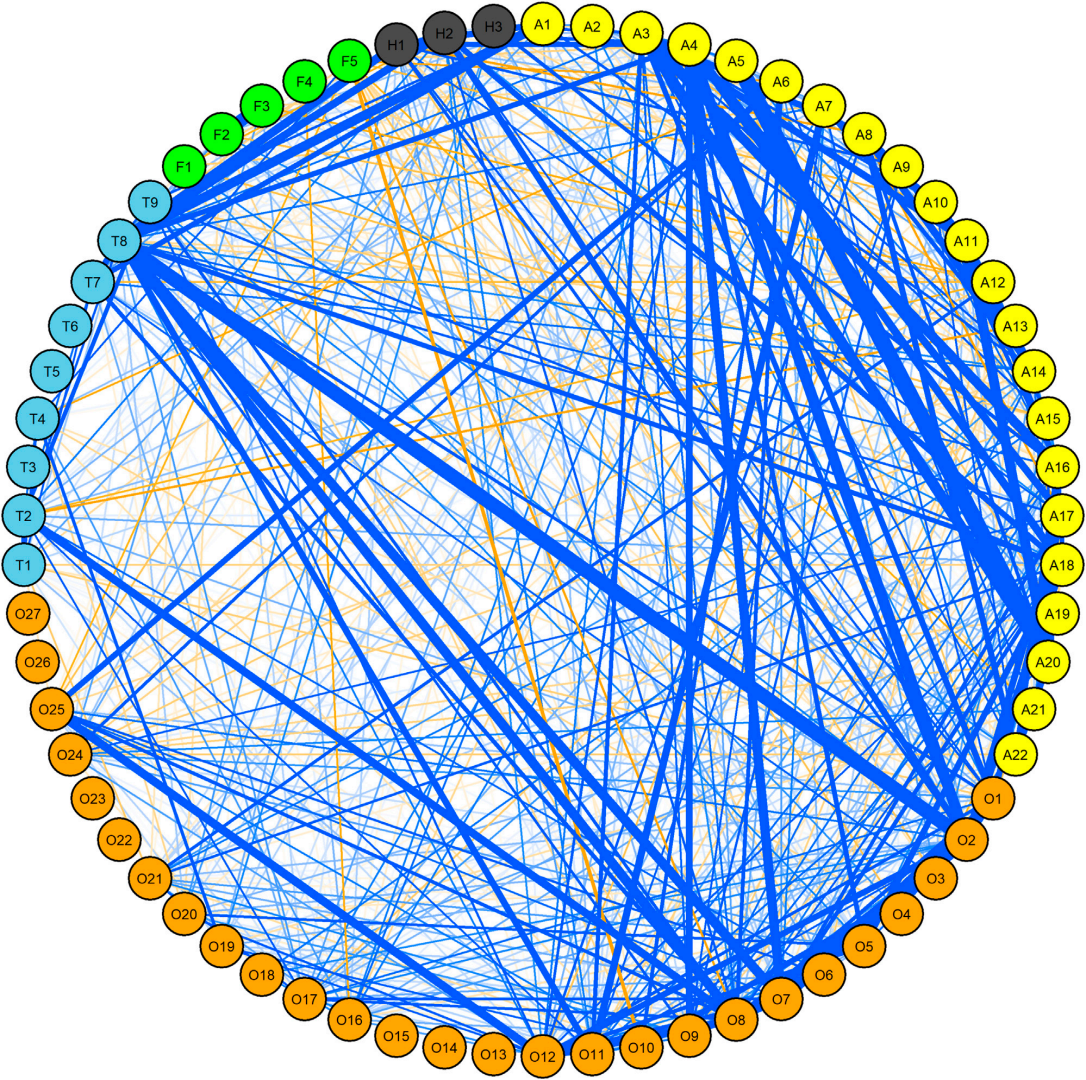
Correlation network among yellow wheat blossom midge (yellow), orange wheat blossom midge (orange), thrip (skyblue), frit fly (green) and seed gall midge (dark gray) in winter wheat (WW) panel. Only correlations > 0.1 R2 and significant at at least 0.05 *p*-values are shown. Blue and orange lines indicate positive and negative correlations, respectively where the thickness of the line is proportional to the strength of the correlation. For details, see Supplementary Table S1.

No definite template existed between various traits with respect to correlation in WW. Some traits of the same insect were, however, in moderate positive correlation (*r* ^2^ > 0.3) (Figure 1, Supplementary Table S3). For example, in the case of OWBM, the correlation of OSL_G12 with OSL_G11, OSL_G13, OSL_G15, and OWSL_G11 as well as OSL_G15 with OSL_G13, OWSA_G13, and OWSA_G13 was >0.3. The maximum *r* ^2^ within OWBM was 0.94 observed between OSL_Q13 and OWSL_Q13. The correlation of YSL_G13 with YWSL_G11, YWSA_G13, YWSL_G15, and YWSL_Q13 as well as YWSA_G13 with YWSL_G11, YWSL_G12, YWSL_G14, YWSL_G15, and YWSA_G15 was >0.3. In the case of thrips, T_G12 and T_G13 were associated with each other at *r* ^2^ = 0.34. The correlation of T_G13 with T_R12 and T_Q16 was 0.35 and 0.33, respectively. Likewise, *r* ^2^ of T_G12 with T_R11 and T_Q15 was 0.3 and 0.32, respectively. T_G11 and T_Q15 were also in moderate positive correlation with *r* ^2^ = 0.38. In the case of FF, the only notable correlation was between F_GS15 and G_GA_14 (*r* ^2^ = 0.59). For SGM, there was a correlation of 0.26 between O12 and O13.

Across years, the correlation between OSL_G11 and YSL_G11, OWSA_G11 and YWSA_G11, OWSL_G11 and OSL_G11, as well as OSL_G11 and T_G11 was >0.3 (Supplementary Table S3). The correlation of YSL_G13 with YWSA_G13, YWSL_Q13, and OSL_G13 was >0.3. Likewise, the correlation of OSL_Q13 with OWSA_G13 was 0.3, and the correlation of OWSA_G13 with OWSL_Q13 and T_G13 was >0.3. There was no notable correlation among traits recorded in 2014. In 2015, YWSL_Q15 and YWSL_G15 were correlated at *r* ^2^ = 0.34. Similarly, OSL_G15 was correlated with OSL_G15 and T_Q15 at 0.32 and 0.49 *r* ^2^, respectively. In 2016, YSL_Q16 and OSL_Q16 were correlated at *r* ^2^ = 0.31.

**FIGURE 2:**
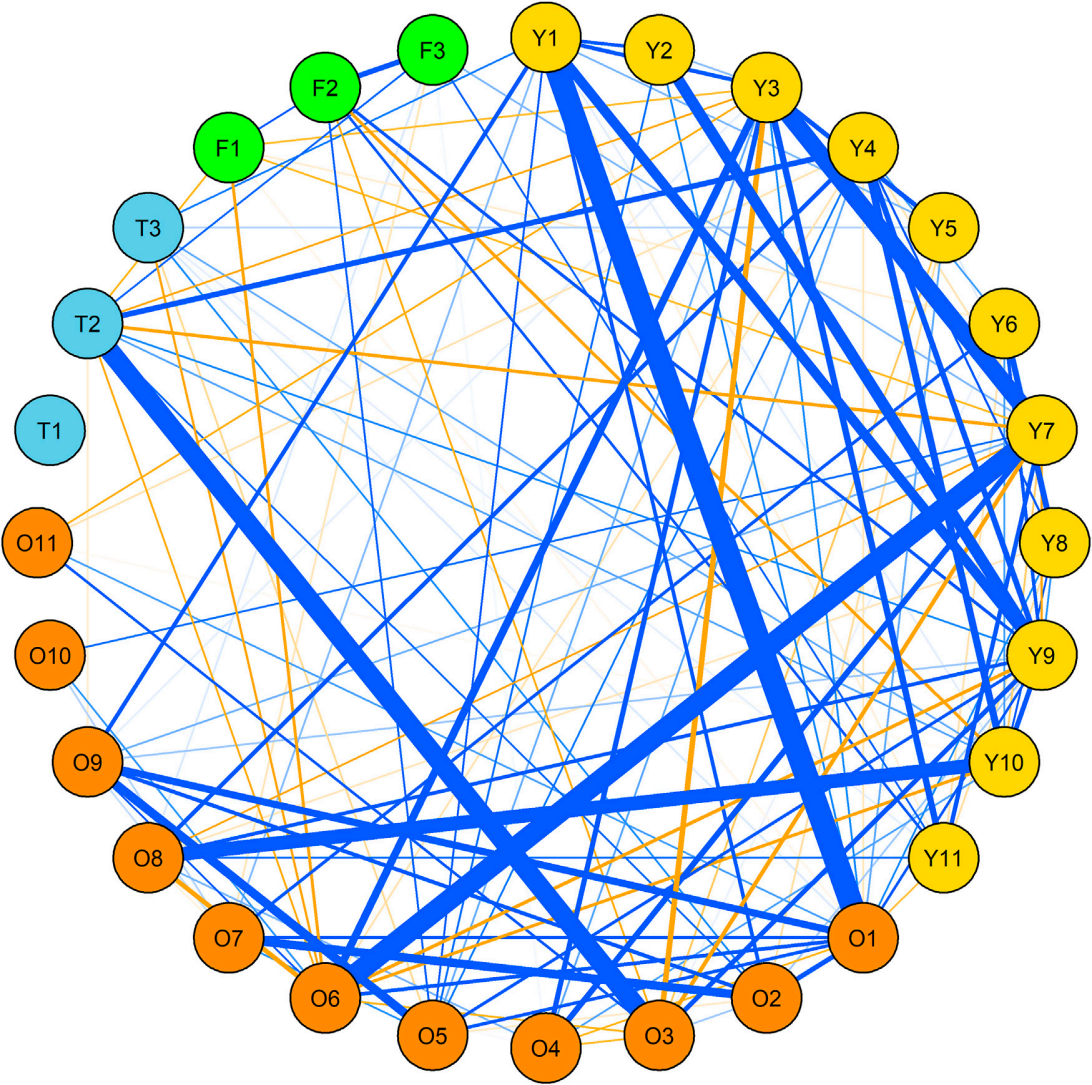
Correlation network among yellow wheat blossom midge (yellow), orange wheat blossom midge (orange), thrip (skyblue) and frit fly (green) in spring wheat (SW) panel. Only correlations > 0.1 R2 and significant at at least 0.05 *p*-values are shown. Blue and orange lines indicate positive and negative correlations, respectively where the thickness of the line is proportional to the strength of the correlation. For details, see Supplementary Table S2.

The correlation pattern did not reveal any specificity among various traits in SW panel as well. The only correlation >0.3 in OWBM traits was between WSL_Q14 and WSA_G12 (*r* ^2^ = 0.33) (Figure 2, Supplementary Table S4). Moreover, among the YWBM traits, the highest *r* ^2^ was 0.53 observed between WSL_G13 and SL_G14. The *r* ^2^ between WSL_G14 and SL_G12 and between WSL_G14 and SL_G13 was 0.35 and 0.39, respectively. All other correlations within YWBM were below 0.3. Likewise, in thrips, the *r* ^2^ between G13 and G15 was 0.28. On the other hand, in the case of FF, the correlation between F_G14 and F_G15 was 0.24.

### Association Analyses

In WW, association analysis of 66 traits examined across a total of 13 environments in Gatersleben (G11, G12, G13, G14, and G15), Quedlinburg (Q12, Q13, Q14, Q15, and Q16), Rosenthal (R12 and R13), and Oberpleichfeld (O13) with 11,139 SNPs (66 × 11,139 = 735,174 data points) provided a total of 645 significant associations (*p*-value < 0.001) where 61 associations were highly significant (*p*-value < 8.98 × 10^−5^) (Supplementary Table S5) (Supplementary Figures S16–S24). Thus, a total of 9.77 SNPs [645 marker-trait associations (MTAs)/66 traits] were associated with one single trait. The distribution of these associations on chromosomes was not uniform. For example, the highest MTAs on one single chromosome was 85 (chromosome 2B) and the lowest was one (chromosome 4D). Among groups, the highest MTAs have detected on group 2 chromosomes (176 MTAs), followed by group 3 chromosomes (138 MTAs) and group 5 chromosomes (101). On the other hand, the lowest number of MTAs has detected on group 4 chromosomes (29 MTAs), followed by group 6 chromosomes (60 MTAs) and group 1 chromosomes (63 MTAs). A total of 78 MTAs were revealed on group 7 chromosomes. Among the genome, the B genome carried the most MTAs (269 MTAs), whereas the D genome carried the least MTAs (123 MTAs). A genome carried 253 MTAs.

From a pest perspective, the highest numbers of MTAs were detected for OWBM (279 MTAs) (Supplementary Figures S16–S18A) followed by YBWM (203 MTAs) (Supplementary Figures S19–S21A). For thrips, we detected 114 MTAs (Supplementary Figure S23A). On the other hand, 20 MTAs (Supplementary Figure S24A) were detected for FF, whereas a total of 29 MTAs were uncovered for SGM (Supplementary Figure S22).

Through the association analysis in SW of 28 traits studied in a total of six environments in Gatersleben (G12, G13, G14, and G15) and Quedlinburg (Q14 and Q15) with 9,804 SNPs (28 × 9,804 = 274, 512 data points), we detected a total of 123 significant MTAs (*p*-value < 0.001) with 11 highly significant associations (*p*-value < 1.019 × 10^−4^) (Supplementary Table S6). The average number of MTAs per trait was 4.39 (123 MTAs/28 traits). Like WW, MTA distribution across the chromosomes was not uniform in SW. For example, there were no MTAs detected on chromosomes 1D, 3D, 4D, 5D, and 6D. The highest numbers of MTAs were detected on group 2 and group 7 chromosomes (26 MTAs each), followed by group 1 chromosomes (25 MTAs) and group 6 chromosomes (16 MTAs). The lowest numbers of MTAs were detected on group 4 chromosomes (seven MTAs), followed by group 5 chromosomes (10 MTAs) and group 3 chromosomes (13 MTAs). B genome carried the most number of MTAs (76 MTAs) followed by A genome (40 MTAs) among the three genomes. D genome carried the least number of seven MTAs.

From insects’ perspective, the highest numbers of MTAs were detected for YWBM (63 MTAs) (Supplementary Figures S19–S21B) followed by OBWM (34 MTAs) (Supplementary Figures S16–S18B). The number of MTAs detected for thrips and FF was 18 (Supplementary Figure S23B) and eight (Supplementary Figure S24B), respectively.

## Discussion

The wheat yield should be increased at the rate of 1.66% against the current rate of 1% per annum to feed the nine billion people by the mid of 21st century. On the other hand, by that time, growing season temperatures will likely exceed those recorded during the 20th century and may substantially reduce crop yields (Deutsch et al., 2018). Crop production losses to pests will increase globally with rising temperatures in all climate models and across all biological parameters. A careful estimate suggests that a 2°C rise in the average global surface temperature will increase the median increase in yield losses due to pest pressure by 46%, causing total estimated losses of up to 59 metric megatons per year. The primary reason for this loss is that warming will increase pest population growth and overwinter survival rates, leading to large population increases in the growing season (Deutsch et al., 2018). It, thus, becomes imperative to develop modern wheat varieties carrying resistant genes against these pests.

### Phenotypic Variation

According to pairwise comparisons, various pests in WW and SW (Supplementary Figures S2, S4, S6, S8, S10, S12, S14) differed mostly in various environments. For example scores of YWBM were higher in G13 and G15 in WW and G12 and G14 in WW and SW, respectively. In OWBM, again, G12 exhibited higher pest attack in SW, whereas the same response in WW was highly variable. Thrips attack was highest in Q15 in both WW and SW, whereas FF attack was comparable in both WW and SW. Weather data (rainfall, number of rainy days, and mean temperature) indicate that the month of May in 2013 was the wettest with 156.6-mm rain in 21 rainy days (Supplementary Table S7) that proved decisive in the considerably higher infection rates of YWBM and OWBM in WW. Before, no such reports exist where a comparison between WW and SW populations was made for any of the mentioned pests. Therefore, comparison in this regard is not possible. Nevertheless, we conclude that both OWBM and YWBM attack differently on WW or SW, which indicates the extraordinary influence of the prevalent environmental conditions before the pest attack. On the other hand, the thrips and FF attack did not differentiate between SW and WW.

### Genetic Analyses

**FIGURE 3:**
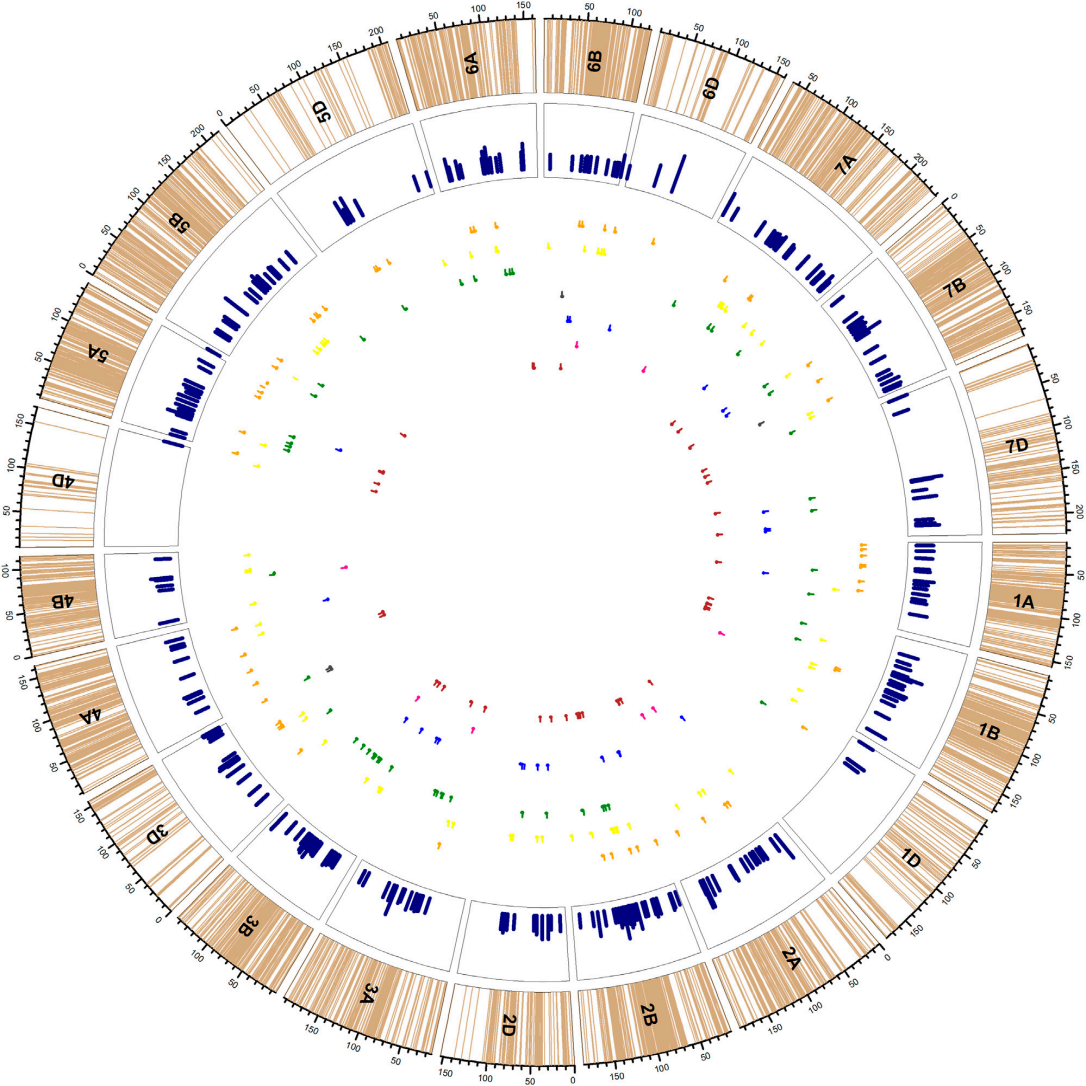
Distribution of 246 QTLs [yellow (yellow wheat blossom midge), orange (orange wheat blossom midge), green (yellow/orange wheat blossom midge), black (saddle gall midge), blue (thrip), pink (frit fly) and maroon (mixed pests)] in the inner circles. Light brown lines in the outer track indicate the SNP positions on each chromosome; pink bars in the second circle indicate the maximum R2 provided by any SNP confided to that QTL. The corresponding lines under the track circle indicate the span of QTLs for respective traits with small vertical lines point to the peak position of QTL.

Because both WW and SW were genotyped with the same SNP chip, we will discuss both the SW and WW simultaneously. On the other hand, for the purpose of discussion, we confined the 645 and 123 MTAs detected in WW and SW, correspondingly, to a total of 246 QTLs (Figure 3, Supplementary Table S8) on the basis of LD among the markers involved in associations (data not shown), following the approach adopted by Dababat et al. (2021). The average span of the QTLs was ∼1.68 cM, whereas the minimum span and maximum span were 1 and 2.76 cM, respectively (on chromosome 5D), that were also variables for the three wheat genomes (1.71, 1.57, and 1.86 cM for A, B, and D genomes, correspondingly). Meanwhile, the average LD decay in WW has been shown to by roughly 5 Mbp, which corresponds to ∼1.2–1.3 cM. The same results were observed in SW population. Real-time LD calculation of the SNPs within the QTLs also indicated that >90% of markers were in absolute LD to each other. The odd one to two SNPs from the cluster of SNPs confined to the QTL were also linked to one of the traits of the main clustered SNPs. We adopted this approach to discuss the genomic regions in association with the traits for a relatively simplified discussion to cater to wide variety of scientists (entomologists and ecologists) who might have relatively less information about the technical details of LD decay and related matters.

Of them, 176 QTLs carried MTAs exclusively detected in WW, 38 QTLs carried MTAs detected exclusively in SW, whereas the 32 QTLs carried MTAs detected both in SW and WW (Table 4), albeit with different insects. From an insect perspective, the numbers of QTLs carrying exclusive QTLs of YWBM, OWBM, thrip, FF, and SGM were 62, 58, 24, 8, and 4, respectively. Another 49 QTLs were related to both YWBM and OWBM (YWBM/OWBM) only. The rest of the 41 QTLs carried at least two or more of the abovementioned pests.

**TABLE 4:** QTL distribution of insect resistance in either winter (WW) or spring (SW) wheat panel or both (WW/SW).

Panel	Trait	Chromosomes																					Total
		1A	1B	1D	2A	2B	2D	3A	3B	3D	4A	4B	4D	5A	5B	5D	6A	6B	6D	7A	7B	7D	
WW	YWBM	1	3	1	1	5	2	**2**	**2**	**3**	**2**	2	1	**1**	**4**	**1**	**3**	**2**		**4**	**2**		42
	OWBM	6	2	1	3	2				**5**	**3**			**5**	**6**	**3**	**3**	**1**	**2**	**2**	**3**		47
	YWBM/OWBM	1	1	1		3	**2**	**5**	4	**2**		1		**3**	**2**	**2**	**2**			**2**	**1**	**2**	34
	Thrips				1	2	**4**	**1**	2		**1**			1				**1**	**1**	**1**	**1**	**2**	18
	FF				1													**1**		**1**			3
	SGM									**2**								**1**			**1**		4
	Other QTLs		3		3	4	2	**2**	1		**1**			4			2	**1**		**1**	**2**	2	28
SW	YWBM		1		3	**2**	2		1			**1**			**2**			**3**		**2**			17
	OWBM	1			1	**2**		**1**			**1**							**1**		**1**			8
	YWBM/OWBM								1												**1**		2
	Thrips	1						**1**										**1**			**1**	**1**	5
	FF		1		1			**1**	1			**1**											5
	Other QTLs					**1**																	1
WW/SW	YWBM											**1**								1	**1**		3
	OWBM					**1**								1				1					3
	YWBM/OWBM	1	1			**1**			3					1	1		2			2	**1**		13
	Thrips					**1**																	1
	Other QTLs	1	2		1	**1**			2		**1**				1						**1**	2	12
		Total	12	14	3	15	25	12	13	17	12	**9**	**6**	**1**	**16**	**16**	**6**	**12**	**13**	**3**	17	**15**	9	246

Bold indicates highly significant association p-value < 0.001 in that QTL on that chromosome.

A total of 58 QTLs of OWBM alone were detected on all the chromosomes except chromosomes 2D, 3B, 4B, 4D, and 7D where the highest numbers of QTLs were located on chromosomes 1A (seven QTLs) and 5A and 5B (six QTLs each) (Supplementary Table S9). The major genes include *laccase-19* [which plays role in the pathogen-induced lignification of secondary cell walls in the rachis (Soni et al., 2020)], *DIBOA-glucoside dioxygenase BX6-like* [wheat BX6 plays role in the formation of benzoxazinoids in planta and contributes to plant resistance against insect herbivores (Shavit et al., 2021)], *CLIP-associated protein-like isoform X2* [CLIP-associated protein 2 (spot 45) is known to be involved in microtubule orientation and stabilization in the plant cell cortex, but the disease/stress responsiveness of this protein is elusive (Ambrose et al., 2007)], *ubiquitin-protein ligase PRT6* and *TOM1-like protein 2* [transporter of mugineic acid (TOM) are important in the maintenance of micronutrient homeostasis (Sharma et al., 2019)], and leucine-rich repeat–containing protein.

A very well-known gene for OWBM resistance, *Sm1*, is known to be located at ∼10- to 13-cM region on the distal portion of chromosome 2BS (Kassa et al., 2016). It was first identified in a collection of WWs from the United States (Thomas et al., 2005). We, however, could not detect *sm1* in our germplasm, probably due to the nature of the germplasm. Although our WW panel includes 20 genotypes that originated from the United States, there was not much difference between the two groups (the United States originated and non-US originated) (data not shown) with regard to OWBM scores. Nevertheless, our germplasm still carried 25 QTLs for insect resistance on chromosome 2B, which is the highest among all chromosomes.

The 62 exclusive QTLs of YBWM were located on all the wheat chromosomes except chromosomes 6D and 7D with the highest number of QTLs on chromosomes 2B and 7A (seven QTLs each). Several candidate genes were identified to be probably involved in YWBM resistance. The major ones include *pentatricopeptide repeat–containing protein* [members of the pentatricopeptide repeat (PPR) protein family are sequence specific RNA binding proteins that play crucial roles in organelle RNA metabolism (Yan et al., 2019)], *tRNA ligase 1 isoform X1*, *chloroplastic glycerophosphodiester phosphodiesterase GDPD1* [GDPDs hydrolyze glycerophosphodiesters into alcohols and glycerol-3-phosphate (G-3-P) suggesting their importance in multiple physiological processes in plants (Nakamura, 2013)], *chlorplastic short-chain dehydrogenase TIC32* [reduces the damage to photosynthetic system upon infection (Hao et al., 2018)], *AP-1 complex subunit sigma-1*, *cytoplasmic iso-leucine-tRNA ligase*, and *transmembrane emp24 domain-containing protein* [transmembrane emp24 domain-containing protein 6-like is recently reported to be an important component of pea aphid saliva-proteome (Caragea et al.)], and many others.

The 49 OWBM/YWBM QTLs were exhibited on chromosomes 1B, 1D, 2B, 2D, 4B, and 6A and all the chromosomes of groups 3, 5, and 7. The major genes located in those regions include *NF-1 related protein kinase regulatory subunit gamma-1–like* [kinases regulate cell growth and proliferation as well as triggering and regulation of immune responses (Theivendren et al., 2021)], *serpin-Z2A-like* [expressed as a fusion protein with the maltose-binding protein (le Roux et al., 2021)], *gamma-secretase subunit APH1-like* [gamma-secretases are localized in the endomembrane of protoplasts in Arabidopsis, and potential role is still unclear (Thomelin, 2018)], *polyubiquitin* and *WPP domain-interacting protein 2* [located on chromosome 5A, this gene is key for nuclear assembly and transport (Gardiner et al., 2019)], *disease resistance protein RGA5-like* and *ankyrin repeat domain-containing protein 2A* [ankyrin repeats are 33-amino-acid sequence motif that are part of protein–protein interaction (Sedgwick and Smerdon, 1999)], *DNA binding protein HEXBP* and *7-deoxyloganetin glucosyltransferase* [∼266 homologous genes belong to 7-deoxyloganetin glucosyltransferase-like gene family (Jiao et al., 2018) which play their role in healing process (Yang et al., 2017) after damage], *protein FAR-RED ELONGATED HYPOCOTYL 3-like* (*FHY3*) [FHY3 and FAR1, two homologous transcription factors are essential for phytochrome A-mediated light signaling (Xie et al., 2020)], and *malate dehydrogenase* [malate dehydrogenases play an important role in central metabolism in plants whose exact role, however, remains unclear (Schreier et al., 2018)].

The use of population independent method (association mapping tool) and different mapping panels allowed us to explore WBM resistance loci in wheat on a very large scale. Our reported QTLs/genes (Supplementary Table S7) have not been reported for insect resistance in wheat before. We, therefore, conclude that these resistance loci can be a potential starting point to impart environment friendly and climate smart WBM resistance in wheat. In addition, arrival of many new technologies such as MACE (Massive Analysis of cDNA 3′ ends) and RNA -sequencing (Duarte-Delgado et al., 2020) may help to understand the mechanisms behind the resistance loci being reported.

Chromosomes 3D, 6B, and 7B carried the four exclusive QTLs for SGM where the most important gene located was *nucleolar GTP-binding protein 1*, which has been reported to act as a positive regulator of stomatal closure in response to both abiotic and biotic stresses (Lee et al., 2017). This result indicates that the candidate gene is involved in the SGM tolerance pathway through its involvement in the stress tolerance defense system. Further molecular genetic investigations are required to understand the mechanism of the candidate gene and if it influences the resistance to other biotic and abiotic stresses.

For thrips exclusively, our germplasm revealed 24 QTLs located on chromosomes 1A, 2A, 2B (three QTLs), 2D (four QTLs), 3A (two QTLs), 3B (two QTLs), 4A, 6B (two QTLs), 6D, 7A, 7B (two QTLs), and 7D (three QTLs). The major genes located in those QTLs include *IAA–amino acid hydrolase ILR1-like 8* [ILR1-like 1 plays its role in metabolic processes resulting in cell growth by releasing IAA through hydrolysis (Du et al., 2017)], *brefeldin A–inhibited guanine nucleotide-exchange protein 1* (*BIG1*) [BIG regulates stomatal immunity and jasmonate production in *Arabidopsis* (Zhang et al., 2019)], and *putative receptor-like kinase*, *serine/threonine-protein kinase*, and *ethylene response factor 1 extended form L* [known to play defense role in various stresses (Arif et al., 2012a)]*.* Therefore, these QTLs are very useful to be involved in breeding programs for improving thrips resistance in wheat that, in turn, increase grain yield and its quality.

The eight exclusive QTLs of FF are located on chromosomes 1B, 2A (two QTLs), 3A, 3B, 4B, 6B, and 7A. The major genes in those QTLs include *nicotinamide/nicotinic acid mononucleotide adenylyltransferase–like* [master enzyme in NAD biosynthesis in living organisms (Zhai et al., 2009)] and *transcriptional corepressor LEUNIG* among the others. The transcriptional corepressor LEUNIG has been reported to be a product of an SNP *Ex_c17379_1431* on chromosome 6B (Suliman et al., 2021). In our case, the SNPs involved were *wsnp_Ex_c17379_26073344* and *RAC875_c17347_312* on chromosome 6B*.* The SNP marker *Ex_c17379_1431* on chromosome 6B coding for the transcription corepressor *LEUNIG* had a significant effect on grain protein content, gluten content, and alveograph strength (Suliman et al., 2021). *LEUNIG* has putative role in the gene regulations in a number of different physiological processes in *Arabidopsis* including disease resistance, DNA damage response, and cell signaling (Gonzalez et al., 2007).

The rest of the 41 QTLs carried MTAs associated with multiple pests. *Fatty acyl-CoA reductase 1* [involved in primary alcohol biosynthesis of the leaf blade cuticular wax in wheat (Wang et al., 2015)], *pentatricopeptide repeat–containing protein* (*PRP*) [PRP proteins are a large family of modular RNA-binding proteins that mediate several aspects of gene expression primarily in organelles but also in the nucleus (Manna, 2015)], *bidirectional sugar transporter SWEET15* [a hormone signaling gene (Gauley and Boden, 2019)], *DNA repair protein rhp54* and *dentin sialophosphoprotein-like* [shell formation protein (Volk et al., 2014)], *villin-4–like* [villin gene family members are associated with multiple stress responses (Lv et al., 2021)], *pyruvate dehydrogenase* [involved in various physiological processes including dormancy, PHS, and seed longevity (Arif et al., 2012a; Arif et al., 2012b)], and *transcriptional corepressor LEUNIG_HOMOLOG* [LEUNIG plays putative role in disease resistance (Gonzalez et al., 2007)].

Our investigation was carried out under natural infection that was under serious environmental influence. Depending on the number of environments in which the pests were scored, we singled out QTLs expressed in multiple environments (for OWBM and YWBM that carried MTAs in three or four different environments and for FF, thrips, and SGM that carried MTAs in two environments).

There were three (on chromosomes 1B, 2B, and 7B) and two QTLs (on chromosomes 3B and 5A) for OWBM that carried MTAs detected in three and four environments, correspondingly. Likewise, there were eight QTLs [on chromosomes 1B, 2A (two QTLs), 2B (three QTLs), 7A, and 7B] that carried multi-environment MTAs discovered in case of YWBM. On the other hand, chromosome 2A and 2B carried QTL with MTAs of FF and SGM from two environments, correspondingly. Furthermore, there were eight chromosomes [chromosomes 2A, 2B (four QTLs), 5B, 6A, and 7B (two QTLs) that carried the multi-environmental MTAs for thrips (Table 5)].

**TABLE 5:** QTLs with SNPs in association with various pests in multiple environments.

QTL name	Chr	Environments	SNPs	Interval	Candidate genes
*Q.OWBM/Thrip/SGM.ipk/jki.1B(WW)*	1B	OWBM (3), T (1), SGM (1)	*Ra_c69552_1419*, *Kukri_c29655_194*, *Ex_c64327_523*, *RAC875_c42715_856*	42.71–43.71	Uncharacterized protein LOC109742350 (*Aegilops tauschii* subsp. strangulata), phosphoinositide phosphatase SAC7-like (*Triticum dicoccoides*)
*Q.OWBM/YWBM/Thrip/FF.ipk/jki.1B(SW/WW)*	1B	YWBM (3), OWBM (2), T(1), FF (1)	*Ku_c11813_215*, *Kukri_c2332_1093*, *Kukri_rep_c101799_95*, *RAC875_rep_c72356_51*, *Kukri_c147_1620*, *BS00090553_51*, *Tdurum_contig61425_242*, *IAAV1158*, *BS00067247_51*	64.6–65.6	Putative clathrin assembly protein At2g01600 (*Aegilops tauschii* subsp. strangulata), pre-mRNA splicing factor SR-like 1 isoform X1 (*Brachypodium distachyon*), UPF0496 protein 4-like [*Triticum dicoccoides*)
*Q.YWBM/OWBM.jki.1B(SW/WW)*	1B	OWBM (1), YWBM (4)	*TA004947_0758*, *BobWhite_c1318_691*, *IACX5764*, *RAC875_c16136_1597*	66.82–67.82	Serine/threonine-protein kinase BSK1-2-like (*Triticum dicoccoides*), putative clathrin assembly protein At2g01600 (*Aegilops tauschii* subsp. strangulata)
*Q.FF.ipk.2A(SW)*	2A	FF (2)	*wsnp_Ex_c2138_4015881*, *Ku_c59581_1412*, *wsnp_Ex_rep_c66615_64916512*	103.7–104.7	Potassium transporter 1 (*Triticum urartu*), protein DETOXIFICATION 16-like (*Triticum dicoccoides*)
*Q.YWBM/Thrip.ipk/jki/ros.2A.4(SW/WW)*	2A	YWBM (3), T (1)	*Tdurum_contig93508_295*, *RAC875_c25848_122*, *IACX5800*, *Tdurum_contig49145_914*, *Ex_c10068_1509*, *Tdurum_contig63071_67*	141–142	Cysteine-rich receptor-like protein kinase 5 (*Triticum dicoccoides*), Putative cyclic nucleotide-gated ion channel 8 (*Triticum urartu*), uncharacterized protein LOC119362705 (*Triticum dicoccoides*)
*Q.Thrip/YWBM/OWBM.ipk/jki.2A(WW)*	2A	OWBM (2), YWBM (2), T (2)	*wsnp_Ex_c10555_17236072*, *Tdurum_contig14482_1073*, *Ex_c36309_435*, *Excalibur_c7971_712*, *IAAV5232*, *IAAV6102*, *IAAV8933*, *Kukri_c25632_86*, *RAC875_c22328_1356*, *RAC875_c22328_490*, *RAC875_c35688_178*, *Tdurum_contig42282_10323*, *Tdurum_contig52350_902*, *Tdurum_contig56321_179*,*BS00024643_51*, *Excalibur_c16329_493*, *Excalibur_c62106_387*, *Kukri_c26697_366*, *RAC875_c35200_230*, *RAC875_c51459_311*, *tplb0025l18_1788*, *BS00098033_51*	151.1–152.1	3-Oxoacyl-[acyl-carrier-protein] synthase 3 B, chloroplastic-like (*Triticum dicoccoides*), hypothetical protein CFC21_020035 (*Triticum aestivum*), hypothetical protein CFC21_026517, partial (*Triticum aestivum*), pentatricopeptide repeat–containing protein At3g53700, chloroplastic-like (*Triticum dicoccoides*), PREDICTED: HBS1-like protein (*Brassica oleracea* var. oleracea), probable leucine-rich repeat receptor-like serine/threonine-protein kinase At3g14840 isoform X3 (*Triticum dicoccoides*), sacsin-like isoform X1 (*Triticum dicoccoides*), dihydroorotate dehydrogenase (quinone), mitochondrial-like (*Triticum dicoccoides*), HBS1-like protein isoform X1 (*Triticum dicoccoides*), Isocitrate and isopropylmalate dehydrogenases family (*Macleaya cordata*), unnamed protein product (*Triticum turgidum* subsp. durum), plastid division protein CDP1, chloroplastic-like (*Triticum dicoccoides*)
*Q.YWBM/OWBM/Thrips.ipk/jki.2A(WW)*	2A	OWBM (2), YWBM (3), T(1)	*CAP8_c3129_381*, *Tdurum_contig10048_207*, *BS00062869_51*	154.5–155.5	LOW QUALITY PROTEIN: endonuclease MutS2-like (*Aegilops tauschii* subsp. strangulata)
*Q.Thrip.jki.2B.1(WW)*	2B	T (2)	*Kukri_c98858_299*, *BobWhite_c7145_355*	24.3–26	Putative disease resistance RPP13-like protein (*Triticum turgidum*)
*Q.Thrip.jki.2B.2(WW)*	2B	T (2)	*Excalibur_c841_609*, *Excalibur_c41459_96*, *Excalibur_c4748_360*, *Kukri_c52200_878*, *RAC875_c2300_1021*	26.8–27.8	Actin-related protein 9-like (Triticum dicoccoides), Hypothetical protein CFC21_014569 (*Triticum aestivum*), unnamed protein product (*Triticum turgidum* subsp. durum)
*Q.Thrip.ipk.2B(SW/WW)*	2B	T (2)	*RAC875_c17720_570*, *wsnp_Ra_c407_862316*	71.1–73.5	NAD-dependent deacetylase sirtuin-6 (*Triticum urartu*)
*Q.YWBM.ipk/jki.2B(WW)*	2B	YWBM (3)	*Ra_c6728_590*, *Ra_c106376_879*, *Kukri_c7139_6288*, *BS00065418_51*	91.1–93.2	Pentatricopeptide repeat–containing protein At4g20740-like (*Triticum dicoccoides*), protein SSUH2 homolog (*Triticum dicoccoides*), Transcription-associated protein 1 (T*riticum urartu*), tRNA ligase 1 isoform X1 (*Aegilops tauschii* subsp. strangulata)
*Q.YWBM/OWBM.ipk/jki.2B.2(SW/WW)*	2B	OWBM (1), YWBM (3)	*Excalibur_c23723_141*, *RAC875_c7827_218*, *BS00041921_51*, *IACX3325*, *RAC875_c46661_184*	94.5–96.5	NF1-related protein kinase regulatory subunit gamma-1–like (*Aegilops tauschii* subsp. strangulata)
*Q.OWBM/Thrip.ipk/jki.2B(SW)*	2B	OWBM (3), T (1)	*RAC875_c36614_344*, *JG_c2092_196*, *Excalibur_c5064_765*, *Excalibur_rep_c67411_210*,*Kukri_c24669_51*, *Kukri_c6552_4243*, *RAC875_c7540_366*, *wsnp_Ex_c1758_3326792*, *wsnp_Ex_rep_c68194_66973531*, *wsnp_Ra_c28955_38371323*, *IAAV3303*, *Tdurum_contig66317_77*	106.8–107.8	Serine racemase (*Elaeis guineensis*), uncharacterized protein LOC119365239 and 119365272 (*Triticum dicoccoides*), 5-amino-6-(5-phospho-D-ribitylamino)uracil phosphatase, chloroplastic-like (*Triticum dicoccoides*), BEACH domain-containing protein C2-like isoform X3 (*Triticum dicoccoides*), hypothetical protein CFC21_031208 (*Triticum aestivum*)
*Q.YWBM/OWBM/Thrip/FF.ipk/jki.2B(WW)*	2B	OWBM (1), YWBM (3), T (2), SGM (2)	*Tdurum_contig54925_225*, *Kukri_rep_c68957_201*, *Ra_c68109_376*, *BS00091068_51*, *wsnp_Ex_c17845_26604587*, *wsnp_Ex_c20182_29230528*, *Tdurum_contig18858_324*, *BobWhite_c5543_492*, *Kukri_c49007_501*, *Kukri_s115194_71*, *BS00077131_51*	109.5–111.5	CNL3 (*Triticum monococcum*), hypothetical protein TRIUR3_01841 (*Triticum urartu*), Kinesin-like protein KIN-7G, partial (*Cucurbita argyrosperma* subsp. sororia), rho GTPase-activating protein 5-like (*Triticum dicoccoides*), villin-4–like (*Triticum dicoccoides*)
*Q.YWBM/OWBM/SGM.ipk/jki/ros.3B(SW/WW)*	3B	OWBM (4), YWBM (2), SGM (1)	*BS00060073_51*, *BS00066467_51*, *BS00073011_51*, *wsnp_Ex_c5547_9774195*, *Ku_c31046_525*, *Ku_c25346_508*, *Kukri_c25794_863*, *tplb0024c09_1335*, *BobWhite_c40455_116*, *BS00091643_51*, *BS00062827_51*, *Excalibur_c15332_1194*, *RAC875_rep_c115516_134*, *Tdurum_contig63110_433*, *BS00097383_51*	73.8–75.5	Transcription factor GAMYB-like (*Triticum dicoccoides*), BAG family molecular chaperone regulator 4 (*Aegilops tauschii* subsp. strangulata), probable LRR receptor-like serine/threonine-protein kinase At2g28960 (*Triticum dicoccoides*), uncharacterized serine-rich protein C1E8.05 (*Aegilops tauschii* subsp. strangulata), probable LRR receptor-like serine/threonine-protein kinase At2g28960 (*Triticum dicoccoides*), dentin sialophosphoprotein-like (*Triticum dicoccoides*)
*Q.YWBM/OWBM.ipk.5A(WW)*	5A	OWBM (4), YWBM (1)	*IAAV1375*, *IAAV3832*	61.2–64.2	Probable UDP-arabinose 4-epimerase 1 (*Sorghum bicolor*), disease resistance protein RGA5-like (*Triticum dicoccoides*)
*Q.YWBM/OWBM/Thrip.ipk/jki.5B(SW/WW)*	5B	OWBM (1), YWBM (2), T (2)	*Tdurum_contig53926_455*, *Tdurum_contig11060_433*, *Kukri_c95103_97*, *wsnp_Ra_c27733_37249132*, *Excalibur_c17055_1451*, *TA001786-1535*, *BobWhite_c16987_106*	69.6–72.1	Hypothetical protein CFC21_073134 (*Triticum aestivum*), cytochrome b561, DM13 and DOMON domain-containing protein At5g54830-like (*Triticum dicoccoides*), unnamed protein product (*Triticum turgidum subsp. durum*), serpin-Z1C (*Triticum dicoccoides*)
*Q.YWBM/Thrip.ipk/jki.6A(WW)*	6A	YWBM (1), T (2)	*BS00109913_51*, *Kukri_c90942_274*, *Tdurum_contig29607_294*	140.7–142.2	Sucrose transport protein SUT4 isoform X2 (*Aegilops tauschii subsp. strangulata*)
*Q.YWBM.ipk/jki.7A(SW/WW)*	7A	YWBM (3)	*Excalibur_c53632_204*, *BS00082180_51*, *CAP7_c10038_214*	130.5–134	
*Q.Thrip.ipk/jki.7B(WW)*	7B	**T (2)**	*GENE_4826_641*, *BobWhite_c10448_80*, *GENE_4337_558*, *Ku_c46689_1653*, *BobWhite_c23074_304*, *BS00003726_51*, *BS00091302_51*	57.8–60	Ethylene response factor 1 extended form L (*Triticum turgidum* subsp. durum), unnamed protein product (*Triticum turgidum* subsp. durum)
*Q.YWBM/OWBM.ipk/jki.7B(WW)*	7B	OWBM (3), YWBM (1)	*Ku_c9598_2119*, *Excalibur_rep_c116920_300*, *Tdurum_contig76683_147*, *wsnp_Ku_c21752_31528824*	72.3–74.8	TBC domain-containing protein C1952.17c isoform X3 (*Aegilops tauschii* subsp. strangulata), serine/threonine/tyrosine protein kinase (*Thinopyrum intermedium*)
*Q.YWBM/Thrips.ipk/jki.7B(WW)*	7B	YWBM (1), T (2)	*RAC875_c68398_75*, *BS00022009_51*, *BS00105558_51*	76.25–79.5	Mitogen-activated protein kinase 12-like (*Triticum dicoccoides*)
*Q.YWBM.ipk.7B(WW)*	7B	YWBM (3)	*RAC875_c8752_1079*, *tplb0045c05_547*	159–160.5	Uncharacterized protein LOC109760071 isoform X2 (*Aegilops tauschii* subsp. strangulata), signal peptide peptidase-like 5 (*Aegilops tauschii* subsp. strangulata)

The use of population independent method (association mapping tool) and different mapping panels allowed us to explore WBM, FF, SGM, and thrips resistance loci in wheat on a very large scale. Our reported QTLs/genes (Supplementary Table S7) have not been reported for insect resistance in wheat before. We, therefore, conclude that these resistance loci can be a potential starting point to impart environment friendly and climate smart insect resistance in wheat. In addition, arrival of many new technologies such as MACE and RNA sequencing (Duarte-Delgado et al., 2020) may help to understand the mechanisms behind the resistance loci being reported.

## Conclusion

All in all, we comprehensively dissected two different wheat germplasm sets for five different wheat pests over a period of 6 years at multiple locations in central Germany. This is the very first report where natural variation in wheat is exploited to map loci linked to YWBM, SGM, FF, and thrips resistance. Moreover, multitude candidate genes are reported of which many are potentially involved in controlling physical structures of plant such as stomatal immunity [*brefeldin A–inhibited guanine nucleotide-exchange protein 1* (*BIG1*)] and closure (*nucleolar GTP-binding protein 1*) and cuticular wax (*Fatty acyl-CoA reductase 1*) of leaf blade to provide physical barriers of insect entry in plants. Others are involved in the production of certain enzymes in response to stress (*DIBOA-glucoside dioxygenase BX6 like* and *villin-4–like*) or play key roles in other physiological processes (*NF-1 related protein kinase regulatory subunit gamma-1–like* and *nicotinamide/nicotinic acid mononucleotide adenylyltransferase-like*). Because this is the first comprehensive report to gauge insect resistance exploiting the natural variation in wheat, the reported SNPs need to be validated. The validation can be achieved by converting reported SNPs into molecular markers applicable felicitous to molecular plant breeding (Cheon et al., 2018) such as KASP (Kompetitive Allele Specific PCR) markers that have successfully been achieved in wheat (Rasheed et al., 2016) for a number of key economic traits. Future research should, therefore, focus on testing this germplasm in other hotspots alongside the development of KASP markers of the reported SNPs for wheat improvement.

## Data Availability

The original contributions presented in the study are included in the article/Supplementary Material. Further inquiries can be directed to the corresponding authors.
