# Supplementary material for: Genetic Insight Into the Insect Resistance in Bread Wheat Exploiting the Untapped Natural Diversity
Source: Front Genet. 2022 Feb 11;13:828905. doi: 10.3389/fgene.2022.828905 (PMC8874221; doi:10.3389/fgene.2022.828905)
Supplement: Supplementary file 1 [file Presentation1.PPTX]

## Slide 1
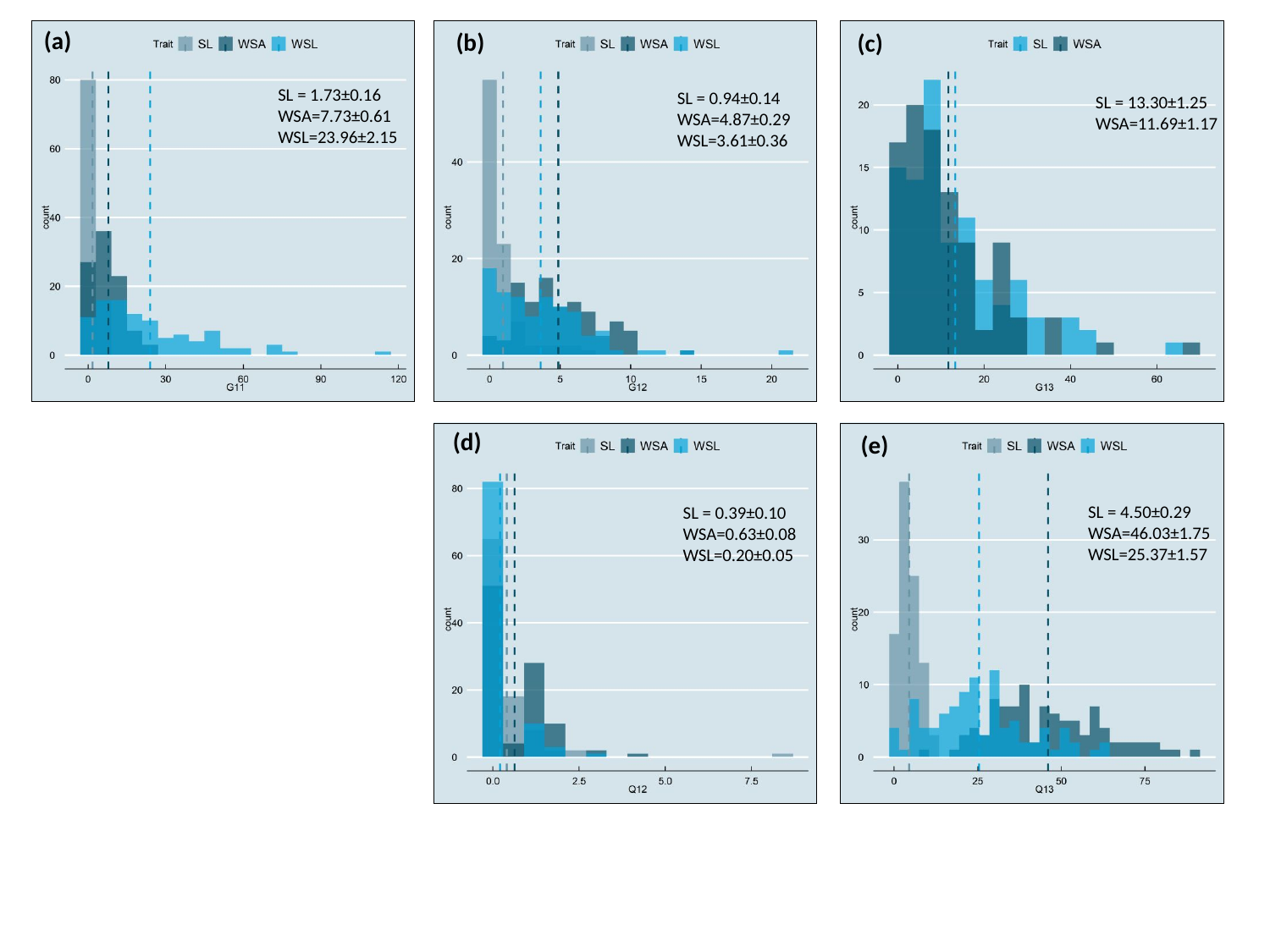

(a)
(b)
(c)
SL = 1.73±0.16
WSA=7.73±0.61
WSL=23.96±2.15
SL = 0.94±0.14
WSA=4.87±0.29
WSL=3.61±0.36
SL = 13.30±1.25
WSA=11.69±1.17
(d)
(e)
SL = 4.50±0.29
WSA=46.03±1.75
WSL=25.37±1.57
SL = 0.39±0.10
WSA=0.63±0.08
WSL=0.20±0.05

## Slide 2
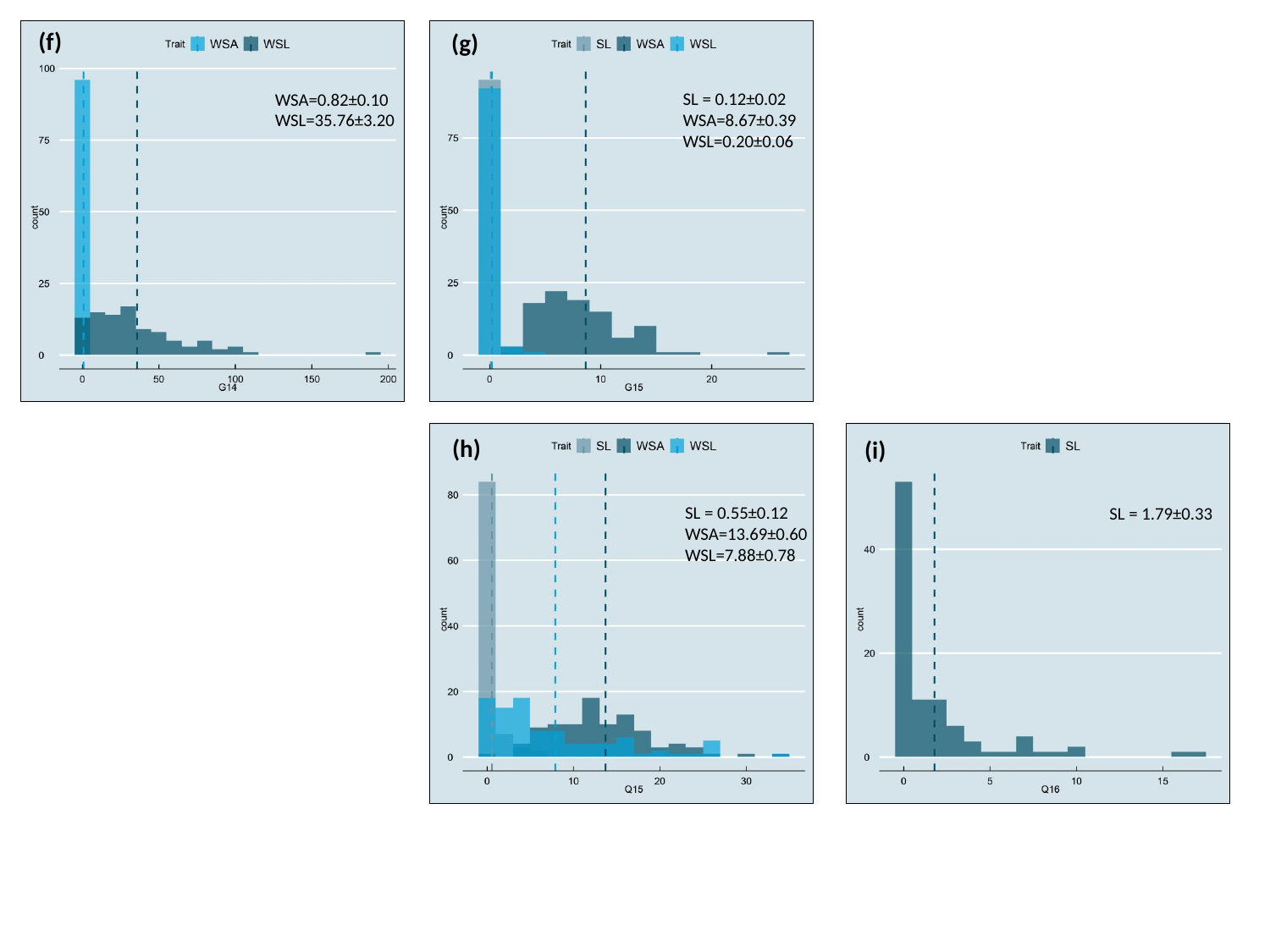

(f)
(g)
SL = 0.12±0.02
WSA=8.67±0.39
WSL=0.20±0.06
WSA=0.82±0.10
WSL=35.76±3.20
(h)
(i)
SL = 0.55±0.12
WSA=13.69±0.60
WSL=7.88±0.78
SL = 1.79±0.33

## Slide 3
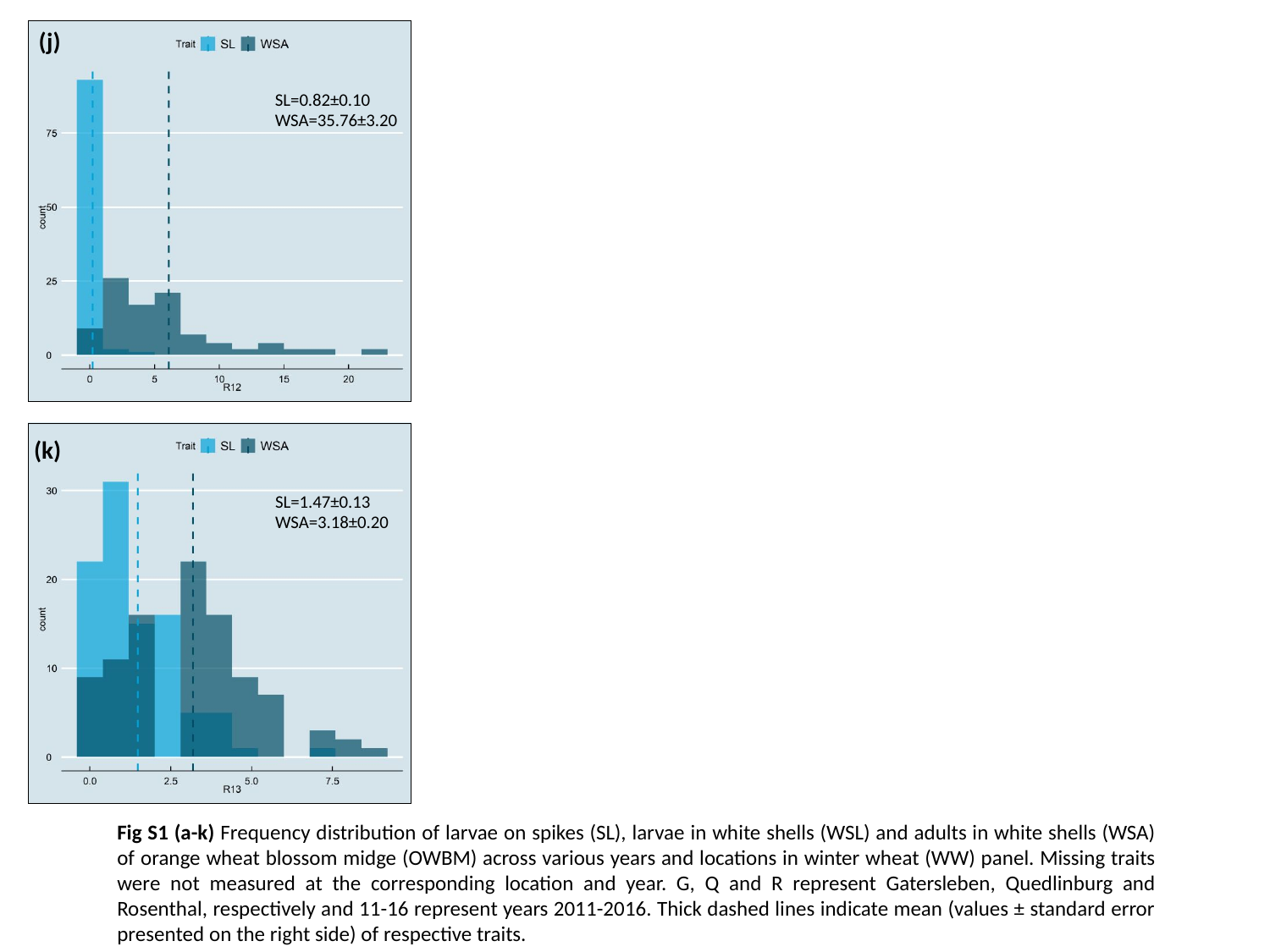

(j)
SL=0.82±0.10
WSA=35.76±3.20
(k)
SL=1.47±0.13
WSA=3.18±0.20
Fig S1 (a-k) Frequency distribution of larvae on spikes (SL), larvae in white shells (WSL) and adults in white shells (WSA) of orange wheat blossom midge (OWBM) across various years and locations in winter wheat (WW) panel. Missing traits were not measured at the corresponding location and year. G, Q and R represent Gatersleben, Quedlinburg and Rosenthal, respectively and 11-16 represent years 2011-2016. Thick dashed lines indicate mean (values ± standard error presented on the right side) of respective traits.

## Slide 4
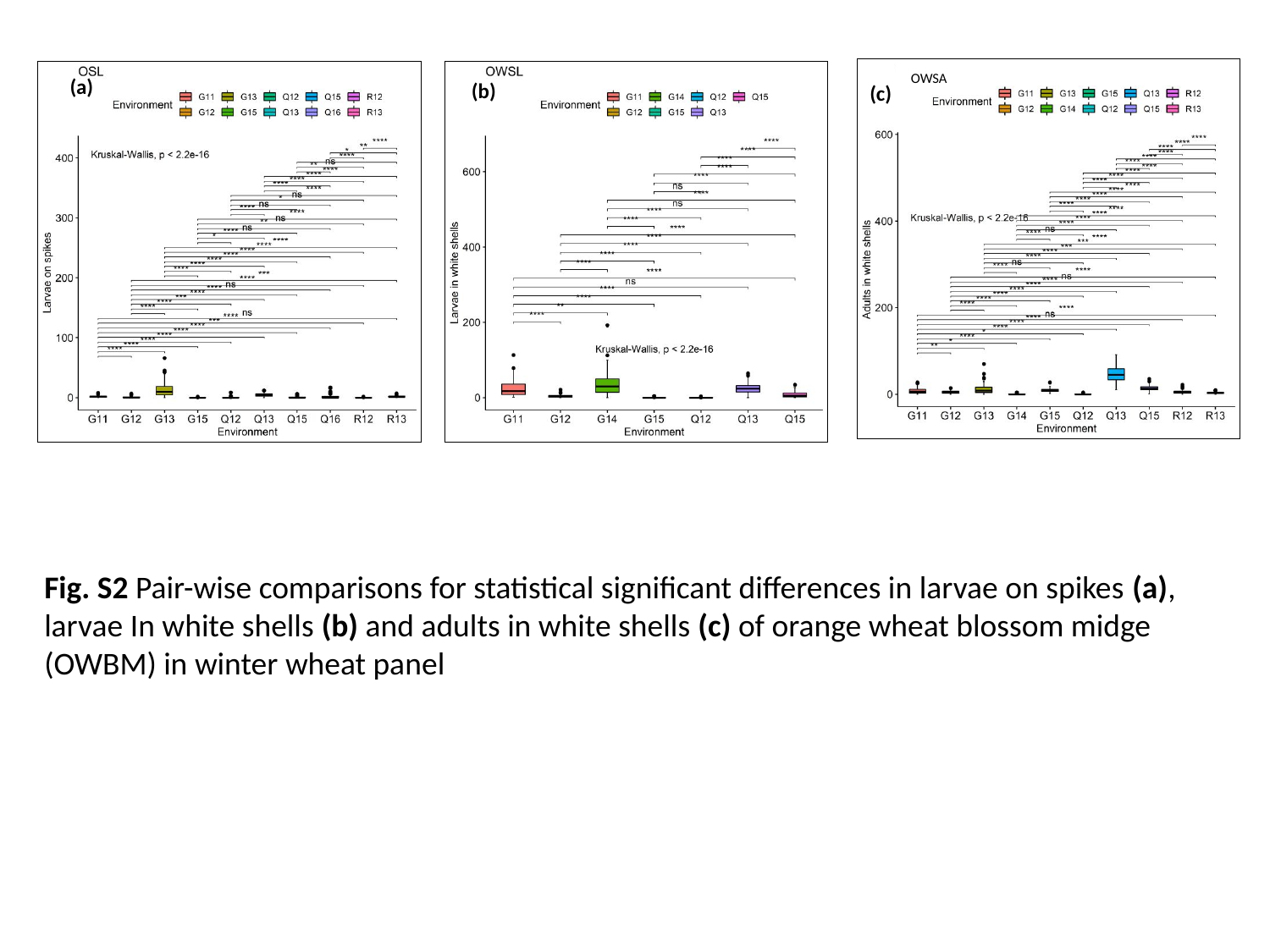

#
OWSA
(a)
(b)
(c)
Fig. S2 Pair-wise comparisons for statistical significant differences in larvae on spikes (a), larvae In white shells (b) and adults in white shells (c) of orange wheat blossom midge (OWBM) in winter wheat panel

## Slide 5
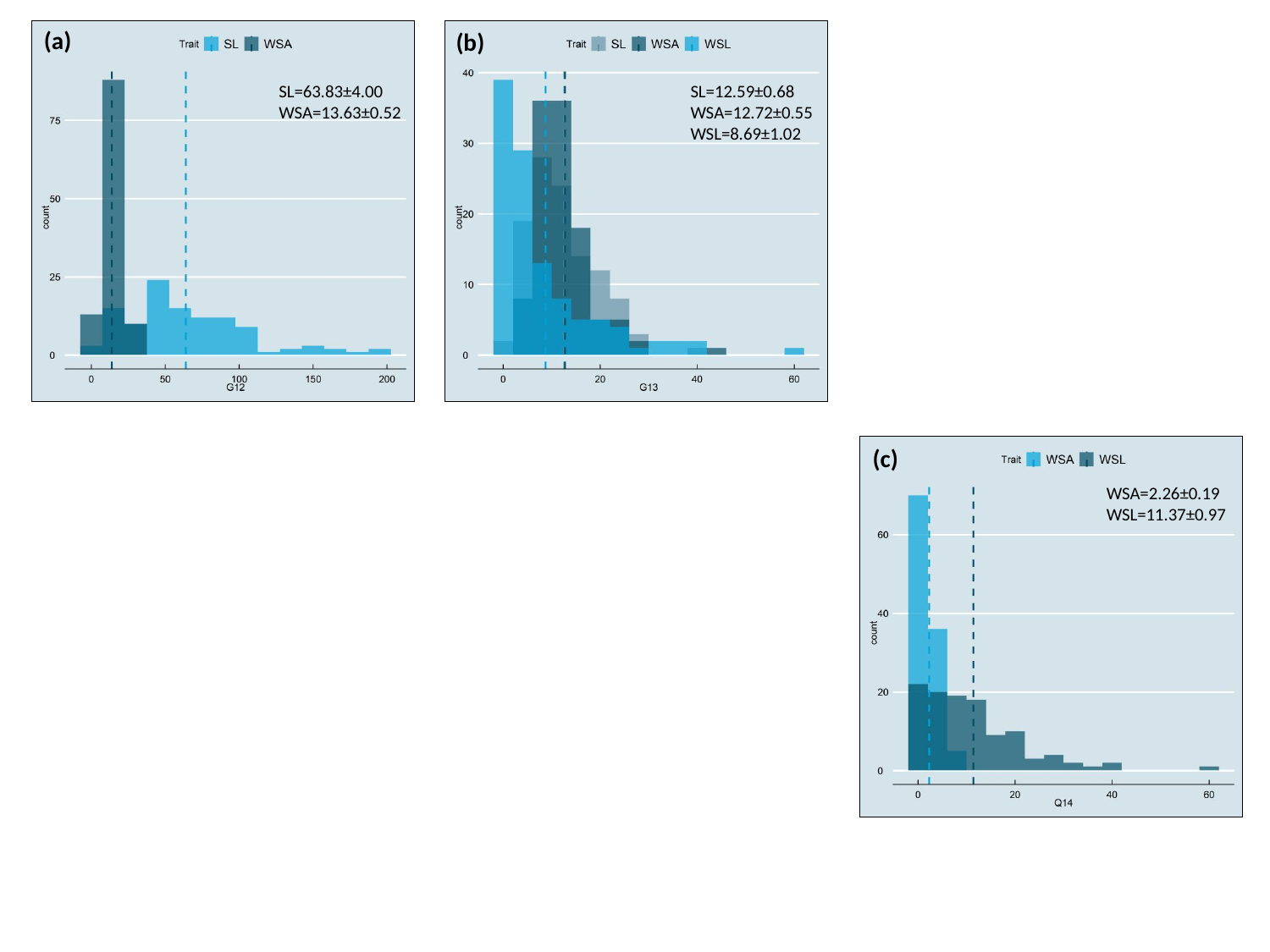

(a)
(b)
SL=63.83±4.00
WSA=13.63±0.52
SL=12.59±0.68
WSA=12.72±0.55
WSL=8.69±1.02
(c)
WSA=2.26±0.19
WSL=11.37±0.97

## Slide 6
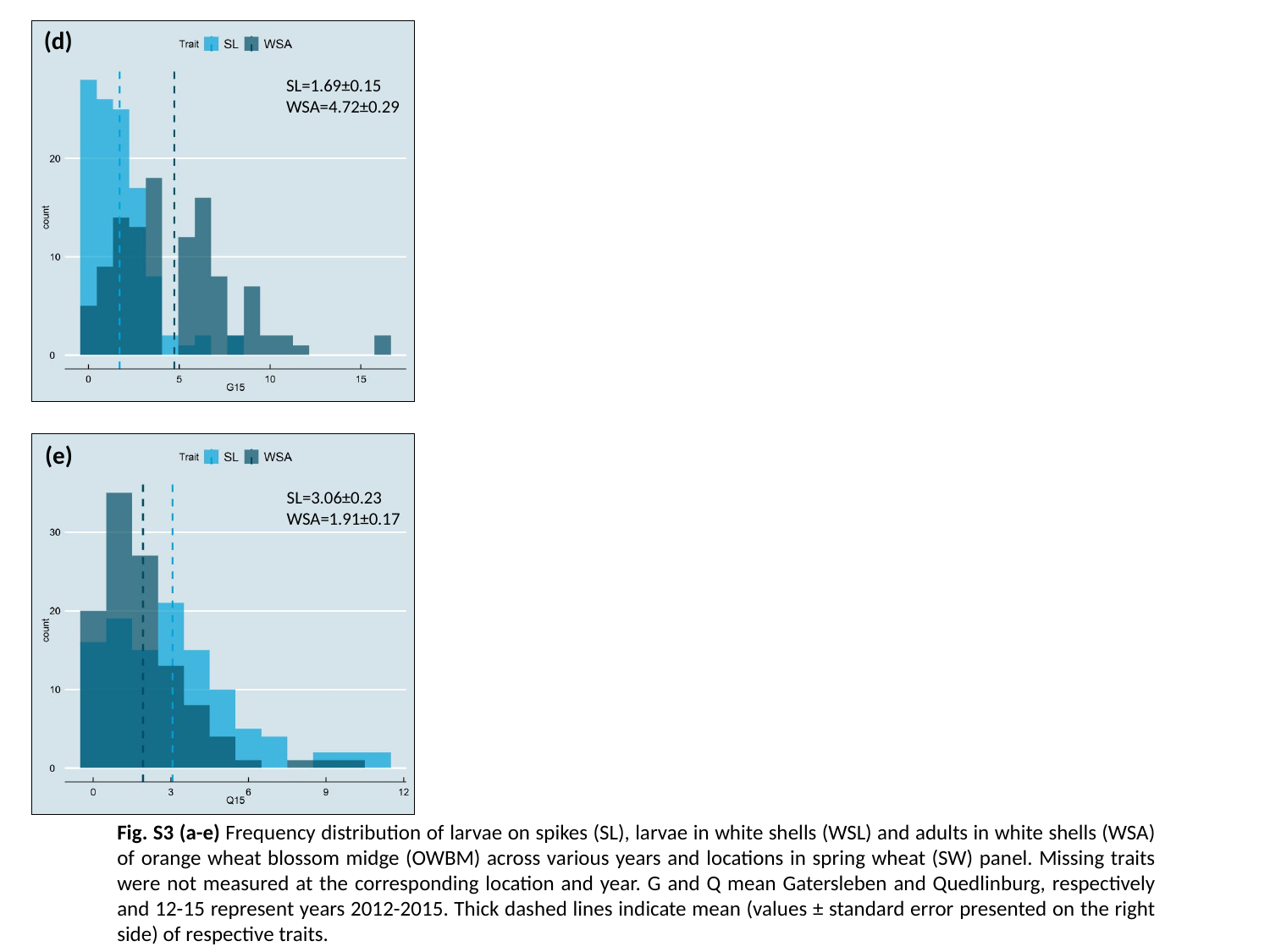

(d)
SL=1.69±0.15
WSA=4.72±0.29
SL=3.06±0.23
WSA=1.91±0.17
(e)
Fig. S3 (a-e) Frequency distribution of larvae on spikes (SL), larvae in white shells (WSL) and adults in white shells (WSA) of orange wheat blossom midge (OWBM) across various years and locations in spring wheat (SW) panel. Missing traits were not measured at the corresponding location and year. G and Q mean Gatersleben and Quedlinburg, respectively and 12-15 represent years 2012-2015. Thick dashed lines indicate mean (values ± standard error presented on the right side) of respective traits.

## Slide 7
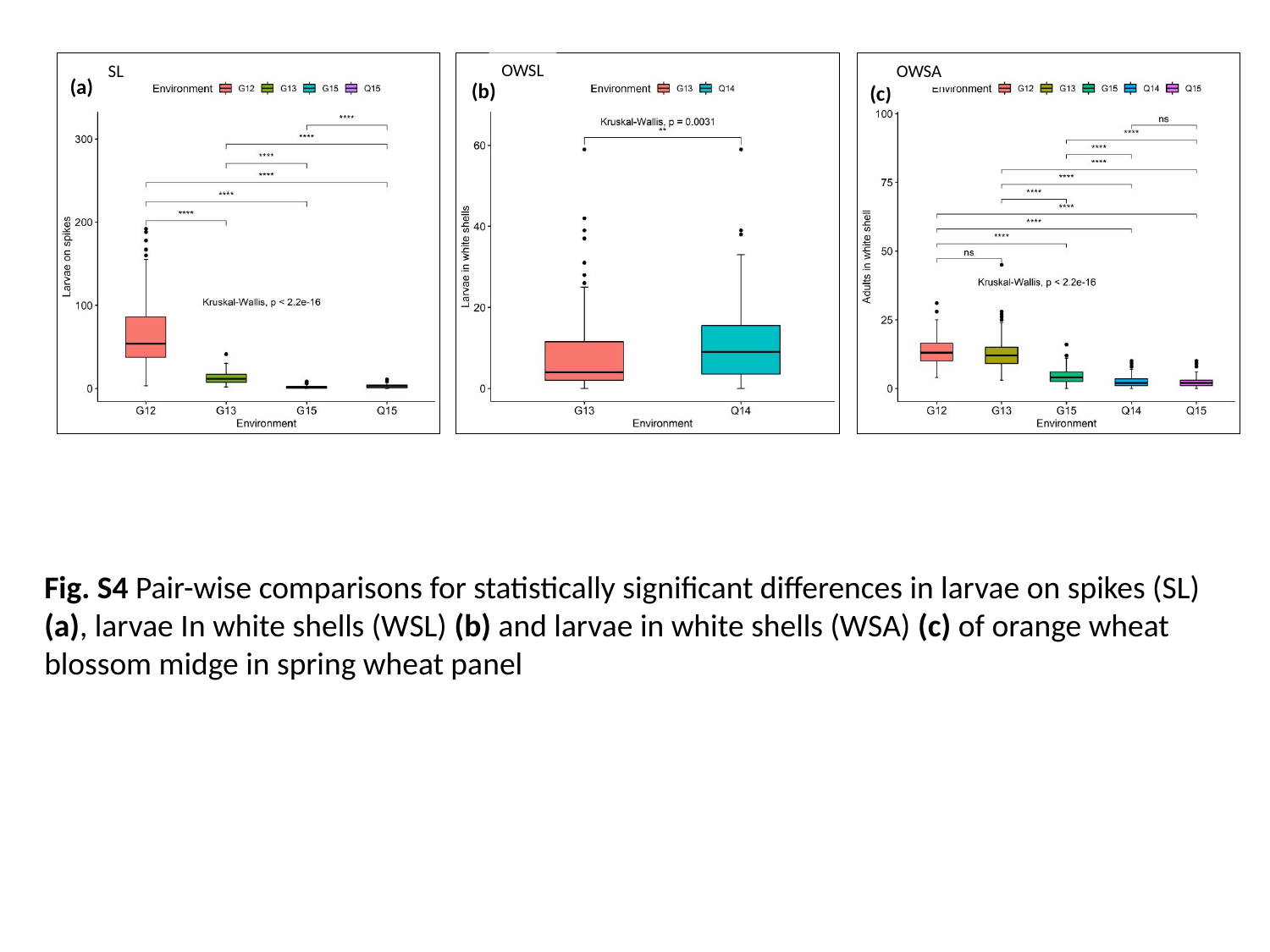

OWSL
OWSA
(a)
(b)
(c)
SL
Fig. S4 Pair-wise comparisons for statistically significant differences in larvae on spikes (SL) (a), larvae In white shells (WSL) (b) and larvae in white shells (WSA) (c) of orange wheat blossom midge in spring wheat panel

## Slide 8
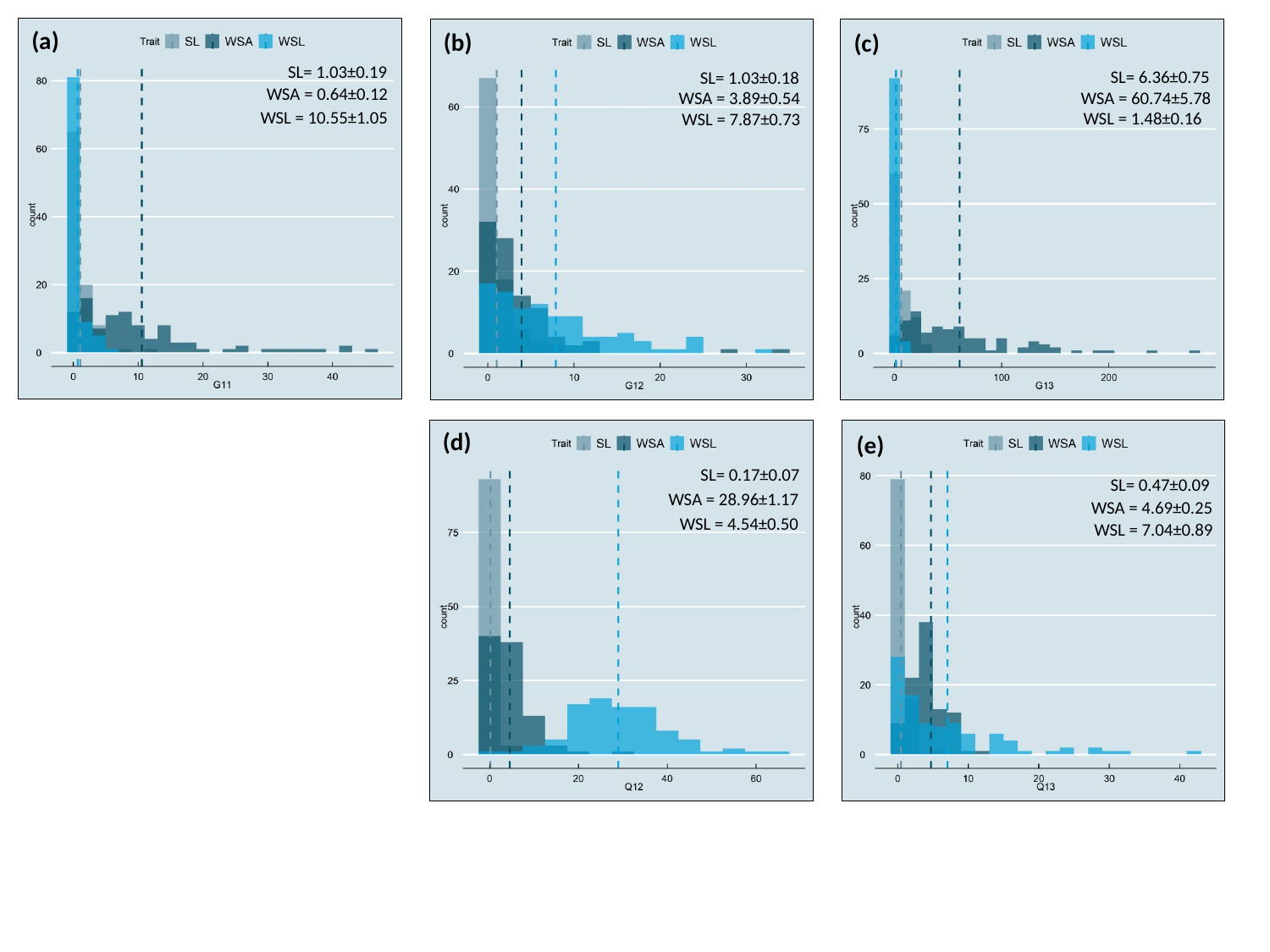

SL= 1.03±0.19
WSA = 0.64±0.12
WSL = 10.55±1.05
(a)
SL= 6.36±0.75
WSA = 60.74±5.78
WSL = 1.48±0.16
SL= 1.03±0.18
WSA = 3.89±0.54
WSL = 7.87±0.73
(b)
(c)
(d)
SL= 0.17±0.07
WSA = 28.96±1.17
WSL = 4.54±0.50
(e)
SL= 0.47±0.09
WSA = 4.69±0.25
WSL = 7.04±0.89

## Slide 9
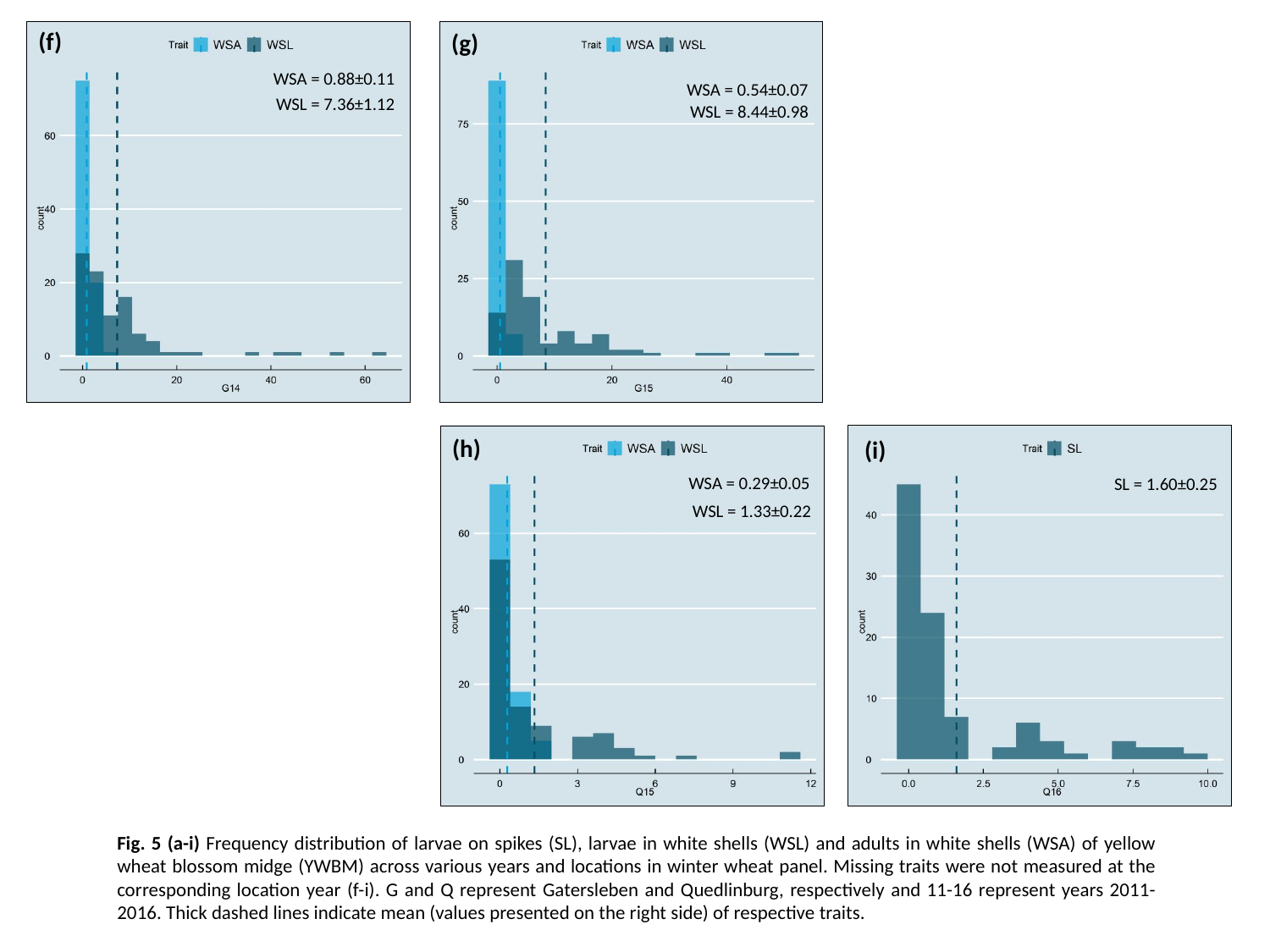

(f)
(g)
WSA = 0.88±0.11
WSL = 7.36±1.12
WSA = 0.54±0.07
WSL = 8.44±0.98
SL = 1.60±0.25
WSA = 0.29±0.05
WSL = 1.33±0.22
(h)
(i)
Fig. 5 (a-i) Frequency distribution of larvae on spikes (SL), larvae in white shells (WSL) and adults in white shells (WSA) of yellow wheat blossom midge (YWBM) across various years and locations in winter wheat panel. Missing traits were not measured at the corresponding location year (f-i). G and Q represent Gatersleben and Quedlinburg, respectively and 11-16 represent years 2011-2016. Thick dashed lines indicate mean (values presented on the right side) of respective traits.

## Slide 10
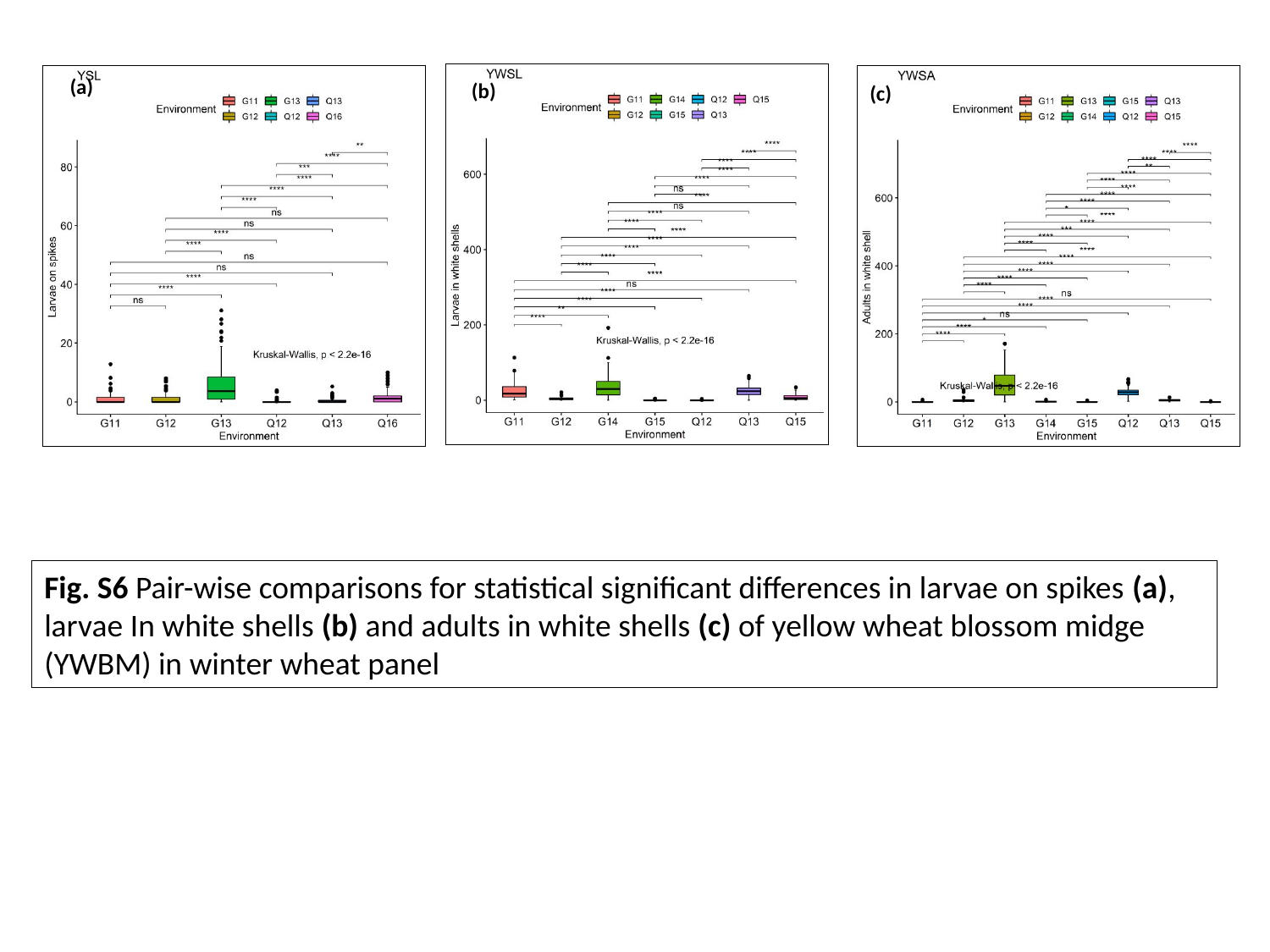

(a)
(b)
(c)
Fig. S6 Pair-wise comparisons for statistical significant differences in larvae on spikes (a), larvae In white shells (b) and adults in white shells (c) of yellow wheat blossom midge (YWBM) in winter wheat panel

## Slide 11
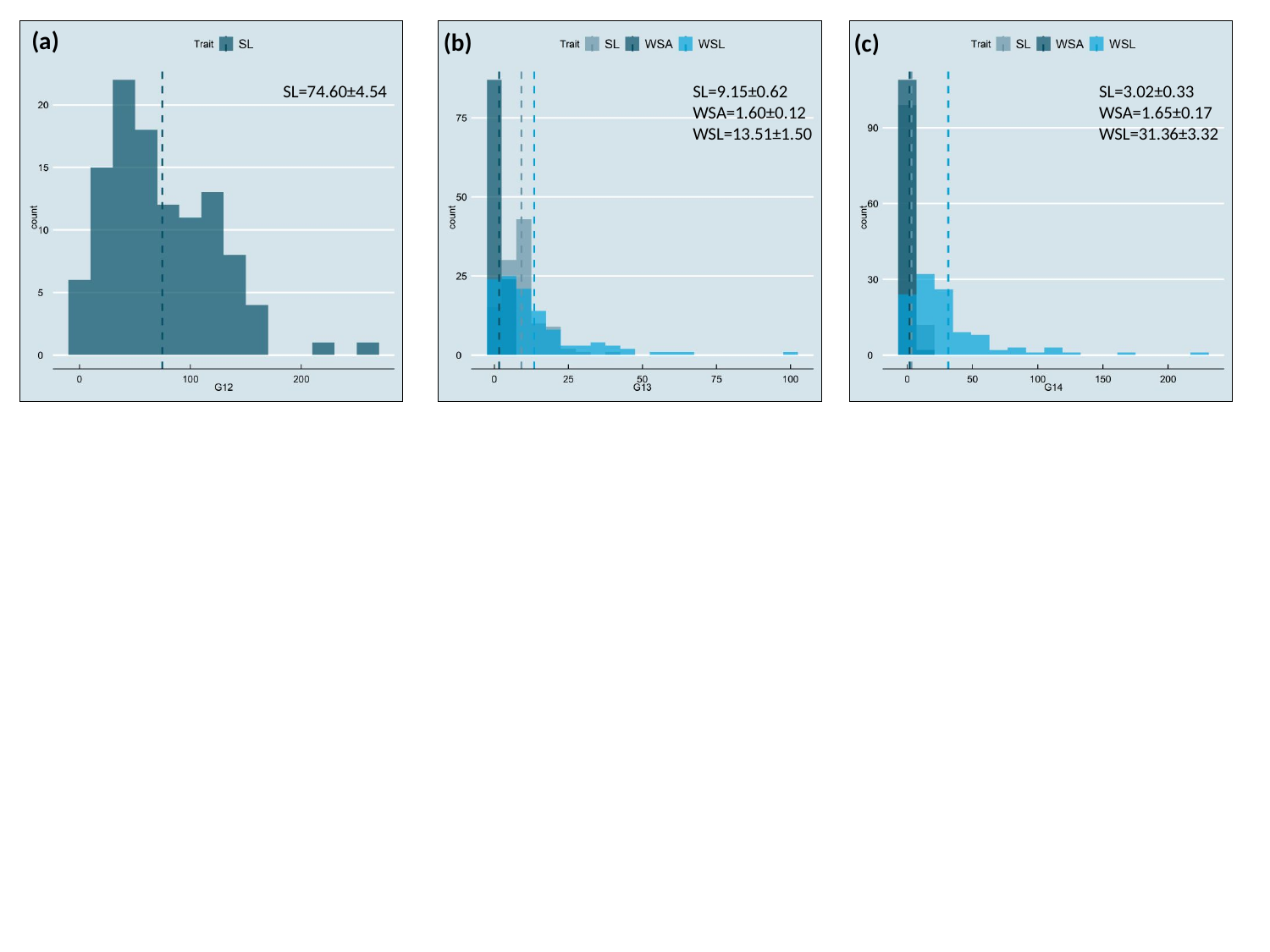

(a)
(b)
(c)
SL=74.60±4.54
SL=9.15±0.62
WSA=1.60±0.12
WSL=13.51±1.50
SL=3.02±0.33
WSA=1.65±0.17
WSL=31.36±3.32

## Slide 12
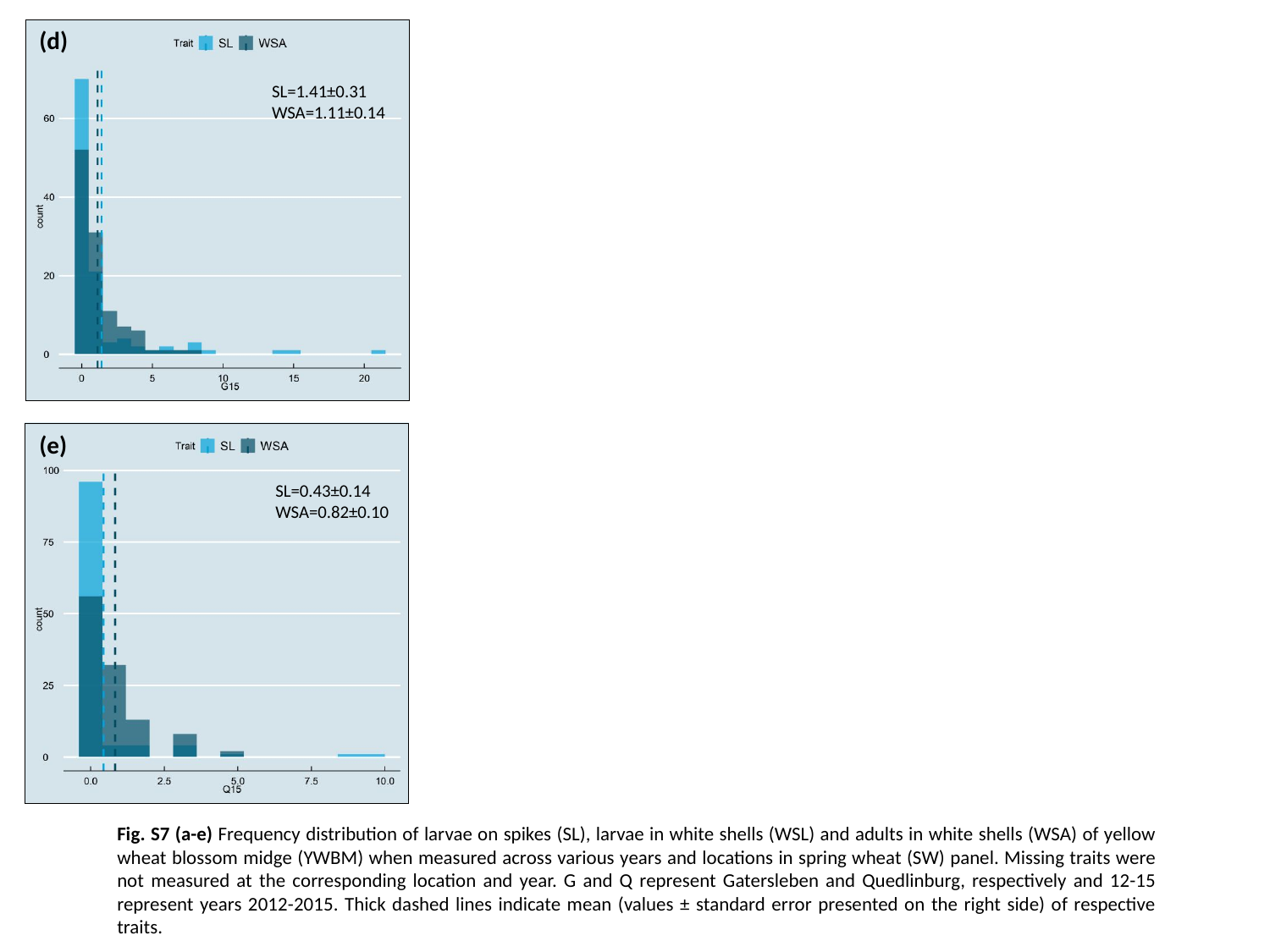

(d)
SL=1.41±0.31
WSA=1.11±0.14
(e)
SL=0.43±0.14
WSA=0.82±0.10
Fig. S7 (a-e) Frequency distribution of larvae on spikes (SL), larvae in white shells (WSL) and adults in white shells (WSA) of yellow wheat blossom midge (YWBM) when measured across various years and locations in spring wheat (SW) panel. Missing traits were not measured at the corresponding location and year. G and Q represent Gatersleben and Quedlinburg, respectively and 12-15 represent years 2012-2015. Thick dashed lines indicate mean (values ± standard error presented on the right side) of respective traits.

## Slide 13
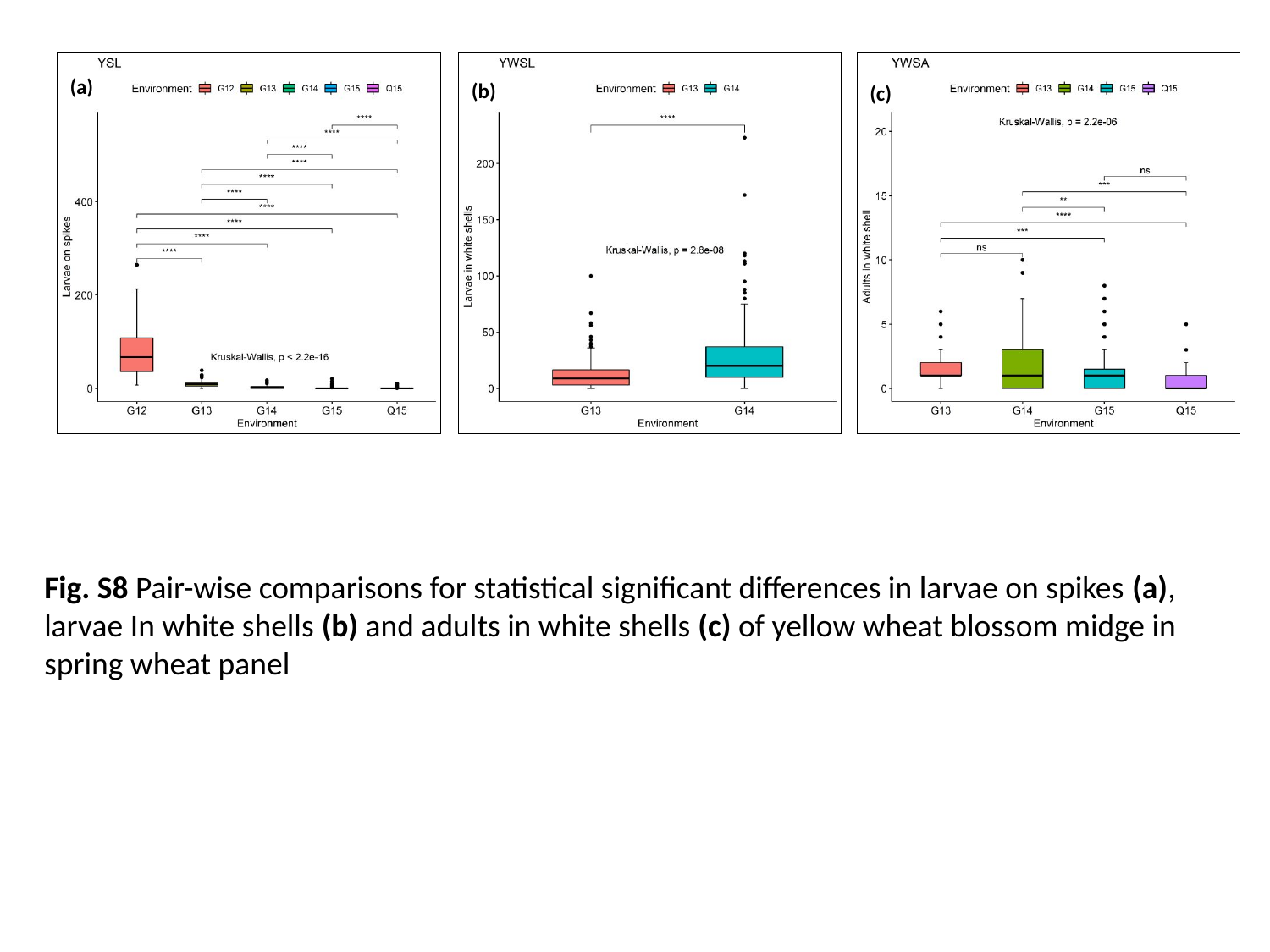

(a)
(b)
(c)
Fig. S8 Pair-wise comparisons for statistical significant differences in larvae on spikes (a), larvae In white shells (b) and adults in white shells (c) of yellow wheat blossom midge in spring wheat panel

## Slide 14
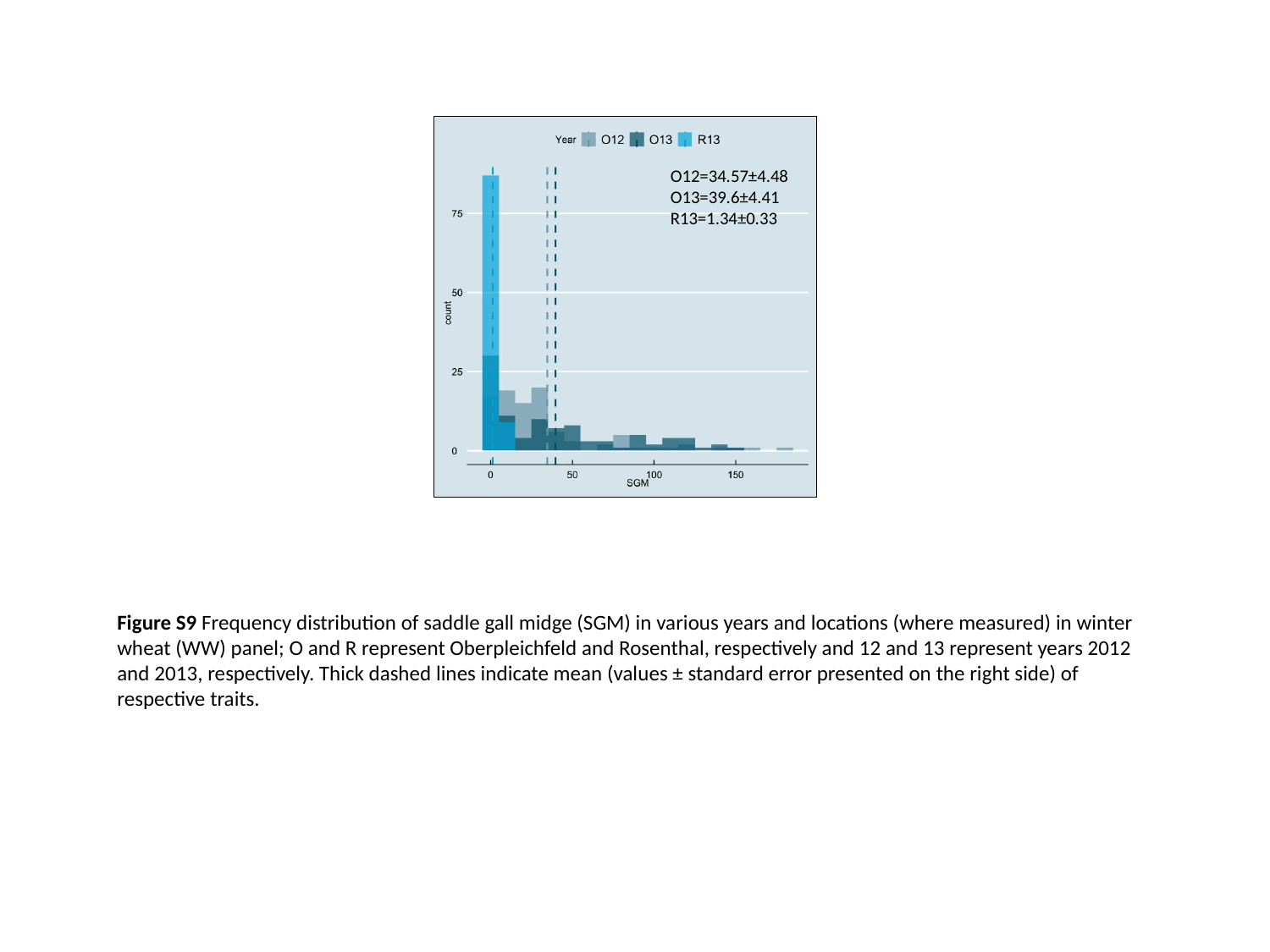

O12=34.57±4.48
O13=39.6±4.41
R13=1.34±0.33
Figure S9 Frequency distribution of saddle gall midge (SGM) in various years and locations (where measured) in winter wheat (WW) panel; O and R represent Oberpleichfeld and Rosenthal, respectively and 12 and 13 represent years 2012 and 2013, respectively. Thick dashed lines indicate mean (values ± standard error presented on the right side) of respective traits.

## Slide 15
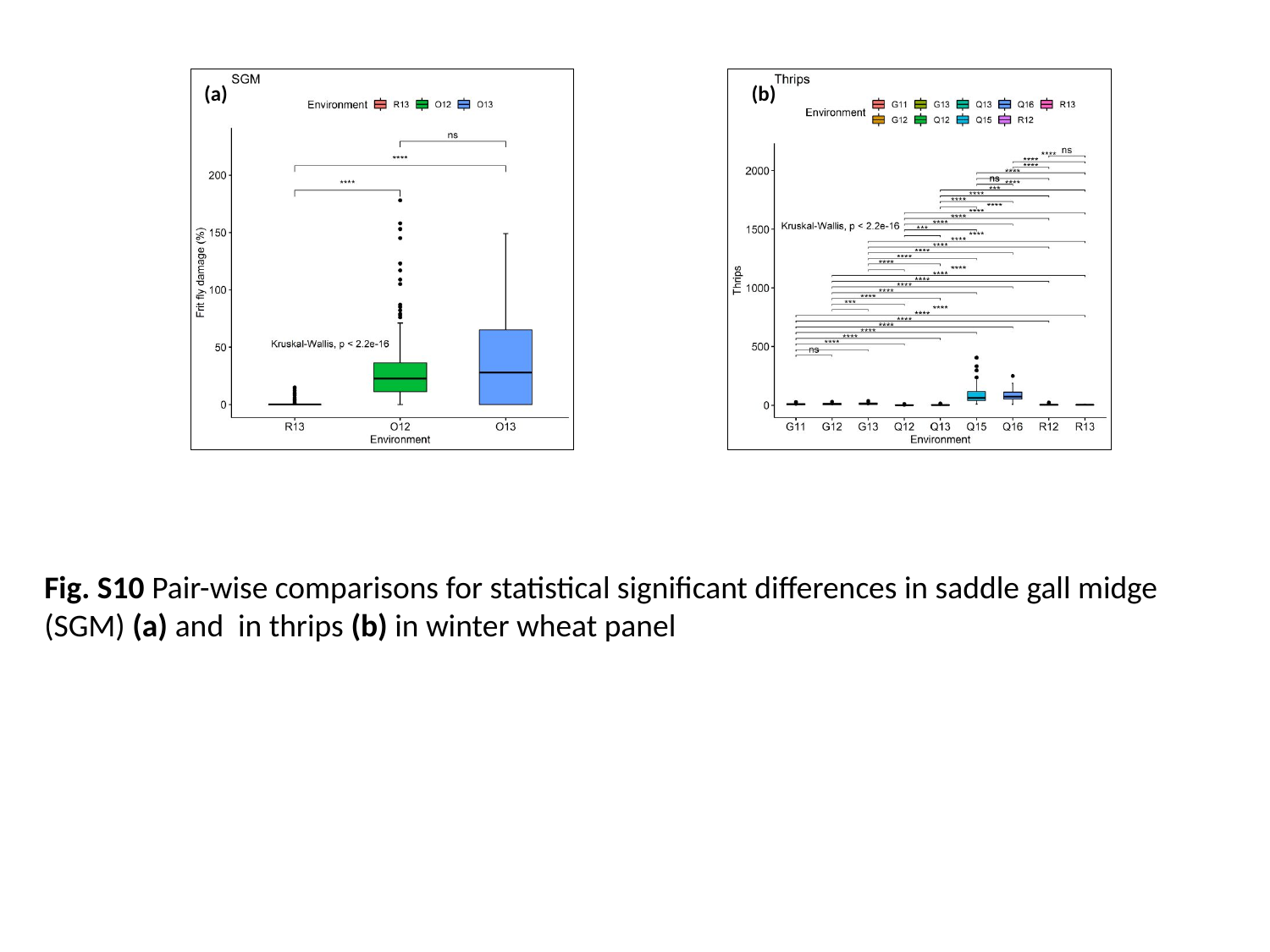

(a)
(b)
Fig. S10 Pair-wise comparisons for statistical significant differences in saddle gall midge (SGM) (a) and in thrips (b) in winter wheat panel

## Slide 16
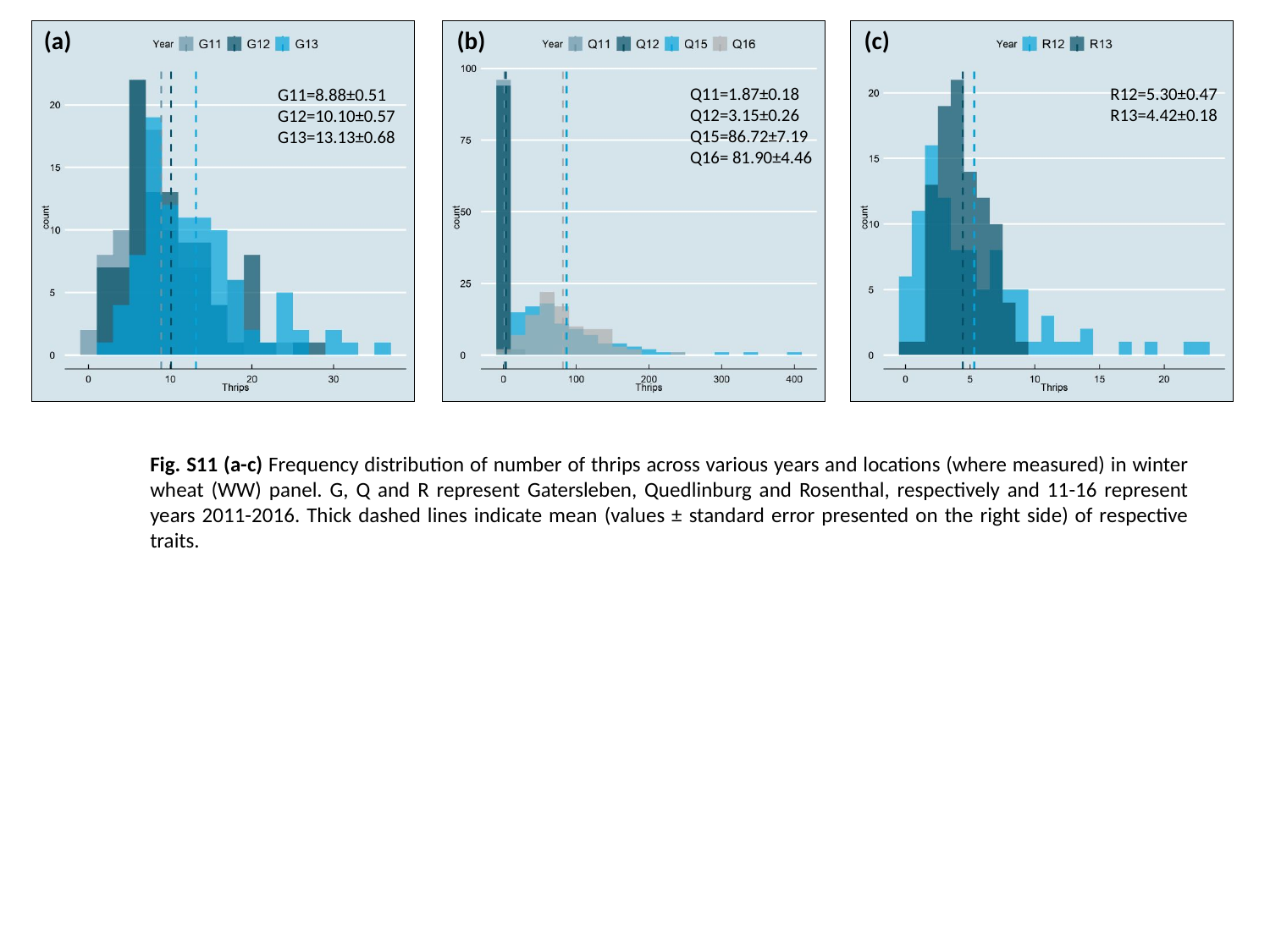

(a)
(b)
(c)
Q11=1.87±0.18
Q12=3.15±0.26
Q15=86.72±7.19
Q16= 81.90±4.46
R12=5.30±0.47
R13=4.42±0.18
G11=8.88±0.51
G12=10.10±0.57
G13=13.13±0.68
Fig. S11 (a-c) Frequency distribution of number of thrips across various years and locations (where measured) in winter wheat (WW) panel. G, Q and R represent Gatersleben, Quedlinburg and Rosenthal, respectively and 11-16 represent years 2011-2016. Thick dashed lines indicate mean (values ± standard error presented on the right side) of respective traits.

## Slide 17
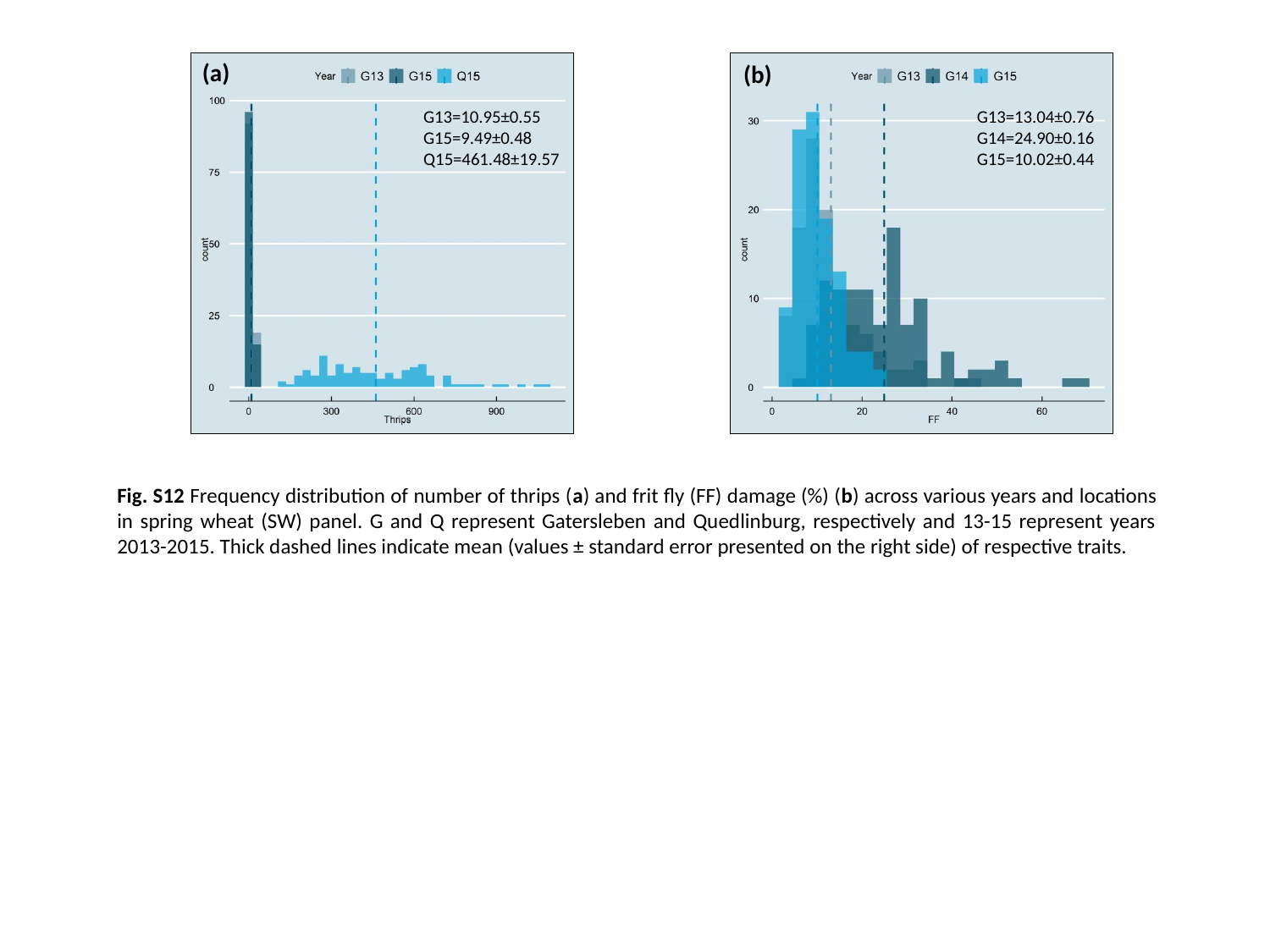

(a)
(b)
G13=10.95±0.55
G15=9.49±0.48
Q15=461.48±19.57
G13=13.04±0.76
G14=24.90±0.16
G15=10.02±0.44
Fig. S12 Frequency distribution of number of thrips (a) and frit fly (FF) damage (%) (b) across various years and locations in spring wheat (SW) panel. G and Q represent Gatersleben and Quedlinburg, respectively and 13-15 represent years 2013-2015. Thick dashed lines indicate mean (values ± standard error presented on the right side) of respective traits.

## Slide 18
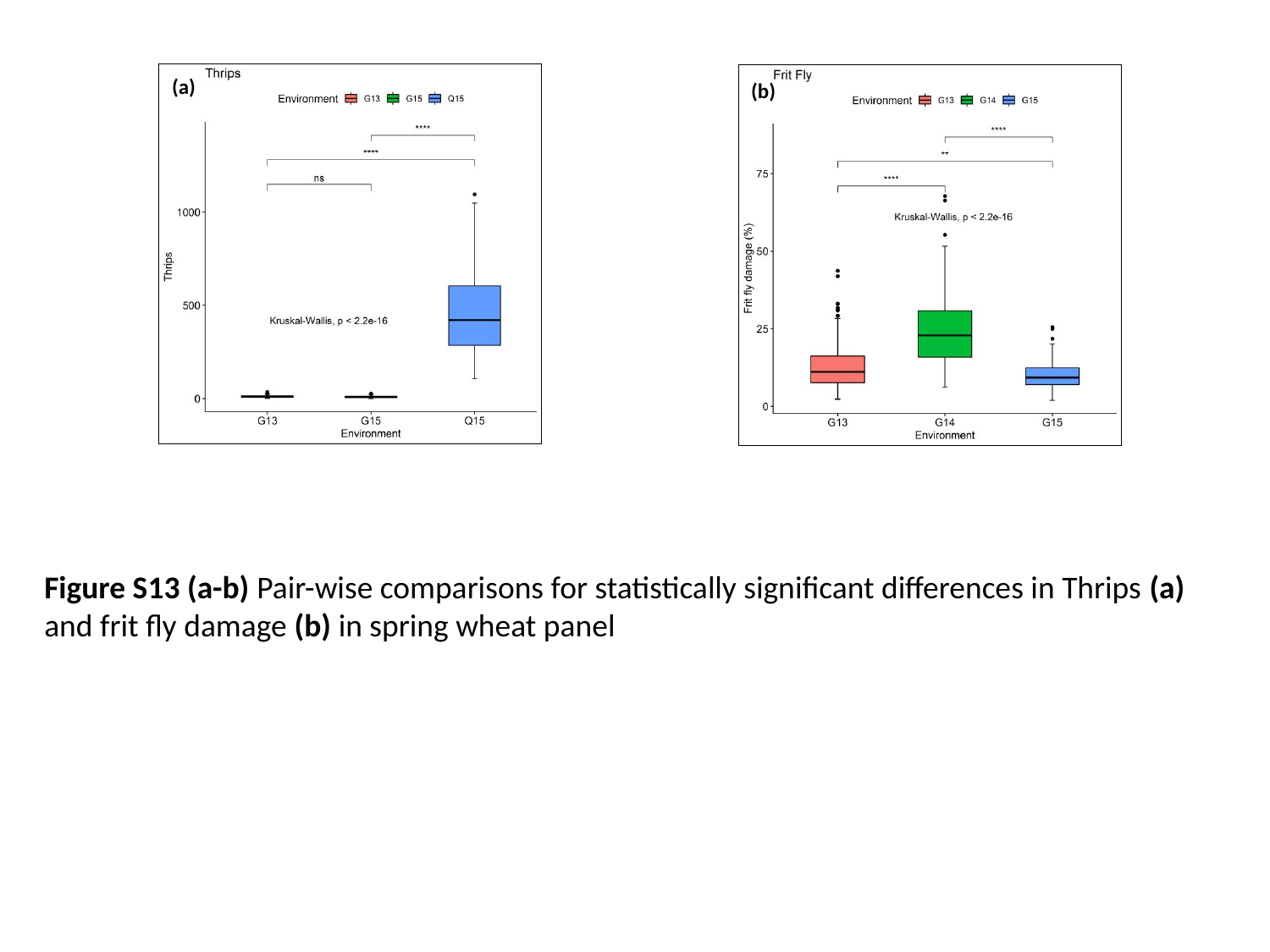

(a)
(b)
Figure S13 (a-b) Pair-wise comparisons for statistically significant differences in Thrips (a) and frit fly damage (b) in spring wheat panel

## Slide 19
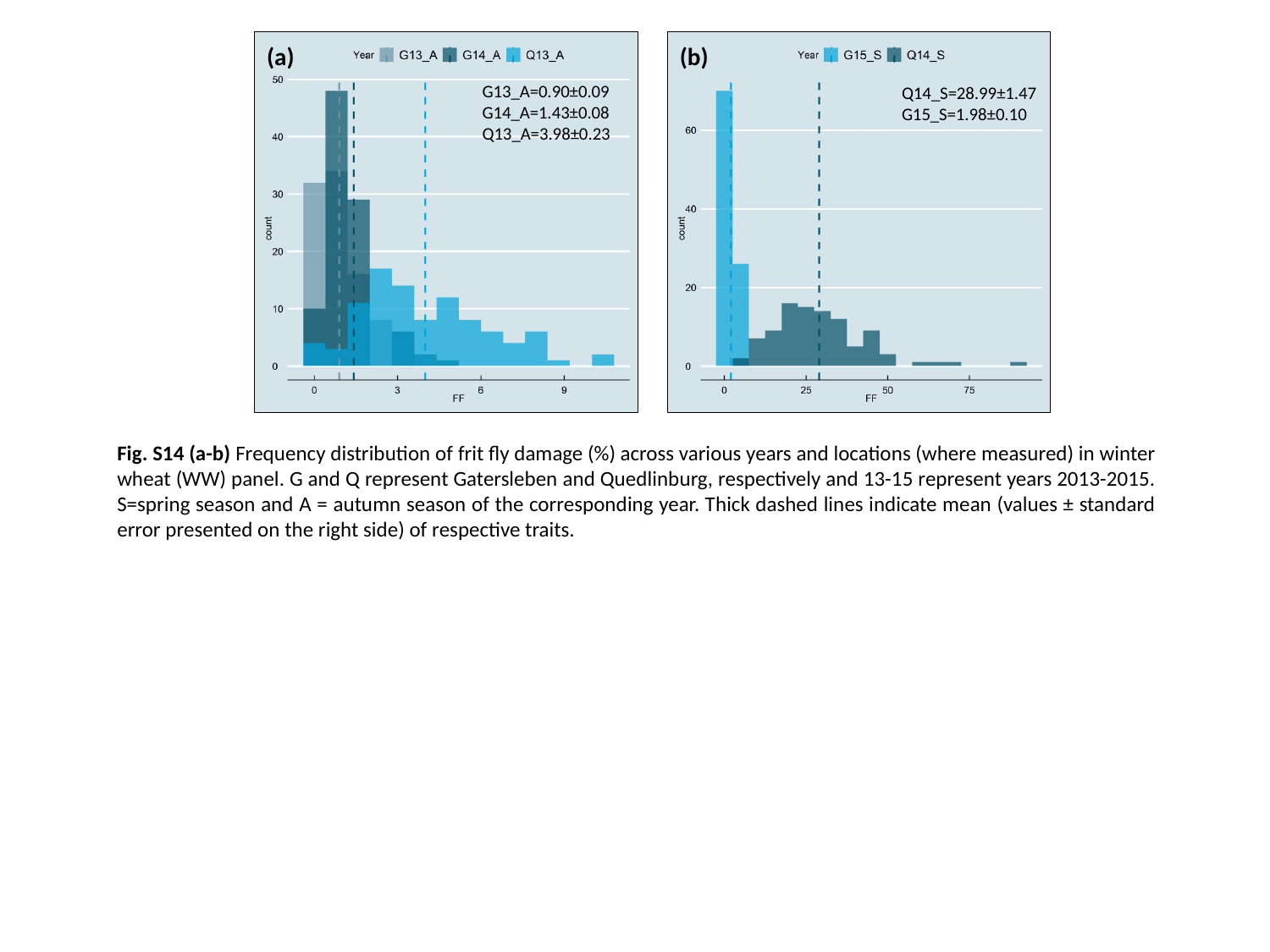

(a)
(b)
G13_A=0.90±0.09
G14_A=1.43±0.08
Q13_A=3.98±0.23
Q14_S=28.99±1.47
G15_S=1.98±0.10
Fig. S14 (a-b) Frequency distribution of frit fly damage (%) across various years and locations (where measured) in winter wheat (WW) panel. G and Q represent Gatersleben and Quedlinburg, respectively and 13-15 represent years 2013-2015. S=spring season and A = autumn season of the corresponding year. Thick dashed lines indicate mean (values ± standard error presented on the right side) of respective traits.

## Slide 20
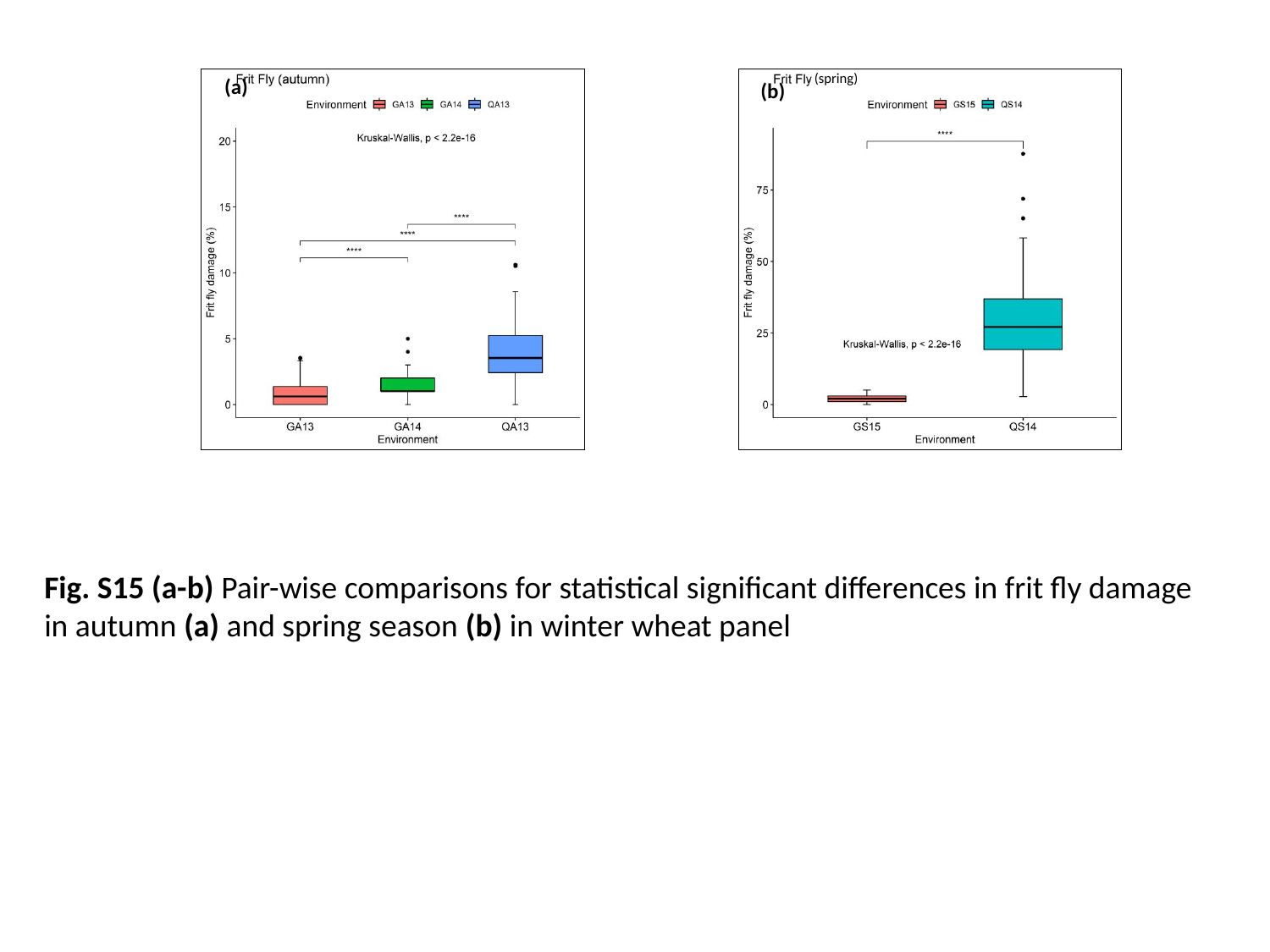

(spring)
(a)
(b)
Fig. S15 (a-b) Pair-wise comparisons for statistical significant differences in frit fly damage in autumn (a) and spring season (b) in winter wheat panel

## Slide 21
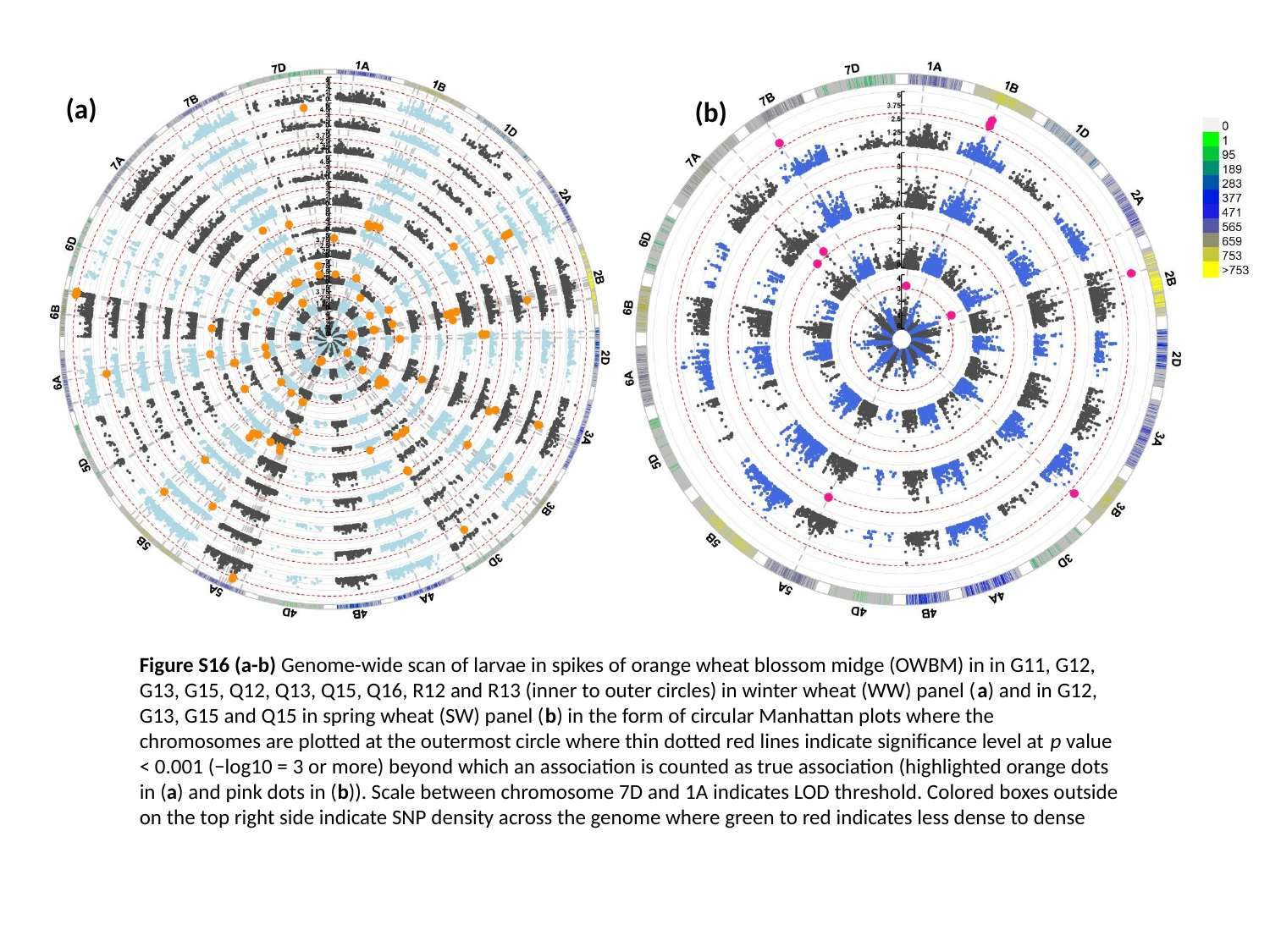

(a)
(b)
Figure S16 (a-b) Genome-wide scan of larvae in spikes of orange wheat blossom midge (OWBM) in in G11, G12, G13, G15, Q12, Q13, Q15, Q16, R12 and R13 (inner to outer circles) in winter wheat (WW) panel (a) and in G12, G13, G15 and Q15 in spring wheat (SW) panel (b) in the form of circular Manhattan plots where the chromosomes are plotted at the outermost circle where thin dotted red lines indicate significance level at p value < 0.001 (−log10 = 3 or more) beyond which an association is counted as true association (highlighted orange dots in (a) and pink dots in (b)). Scale between chromosome 7D and 1A indicates LOD threshold. Colored boxes outside on the top right side indicate SNP density across the genome where green to red indicates less dense to dense

## Slide 22
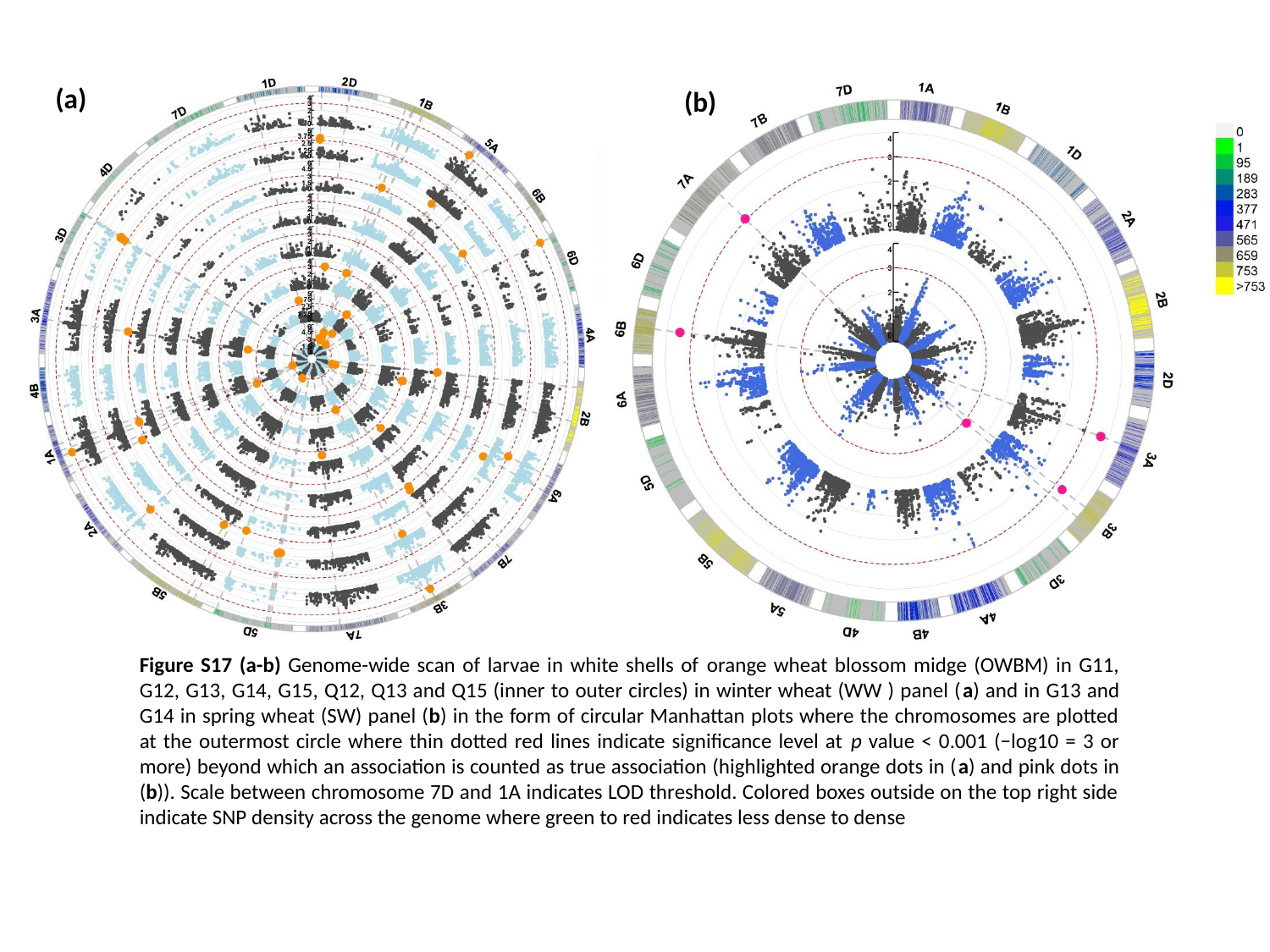

(a)
(b)
Figure S17 (a-b) Genome-wide scan of larvae in white shells of orange wheat blossom midge (OWBM) in G11, G12, G13, G14, G15, Q12, Q13 and Q15 (inner to outer circles) in winter wheat (WW ) panel (a) and in G13 and G14 in spring wheat (SW) panel (b) in the form of circular Manhattan plots where the chromosomes are plotted at the outermost circle where thin dotted red lines indicate significance level at p value < 0.001 (−log10 = 3 or more) beyond which an association is counted as true association (highlighted orange dots in (a) and pink dots in (b)). Scale between chromosome 7D and 1A indicates LOD threshold. Colored boxes outside on the top right side indicate SNP density across the genome where green to red indicates less dense to dense

## Slide 23
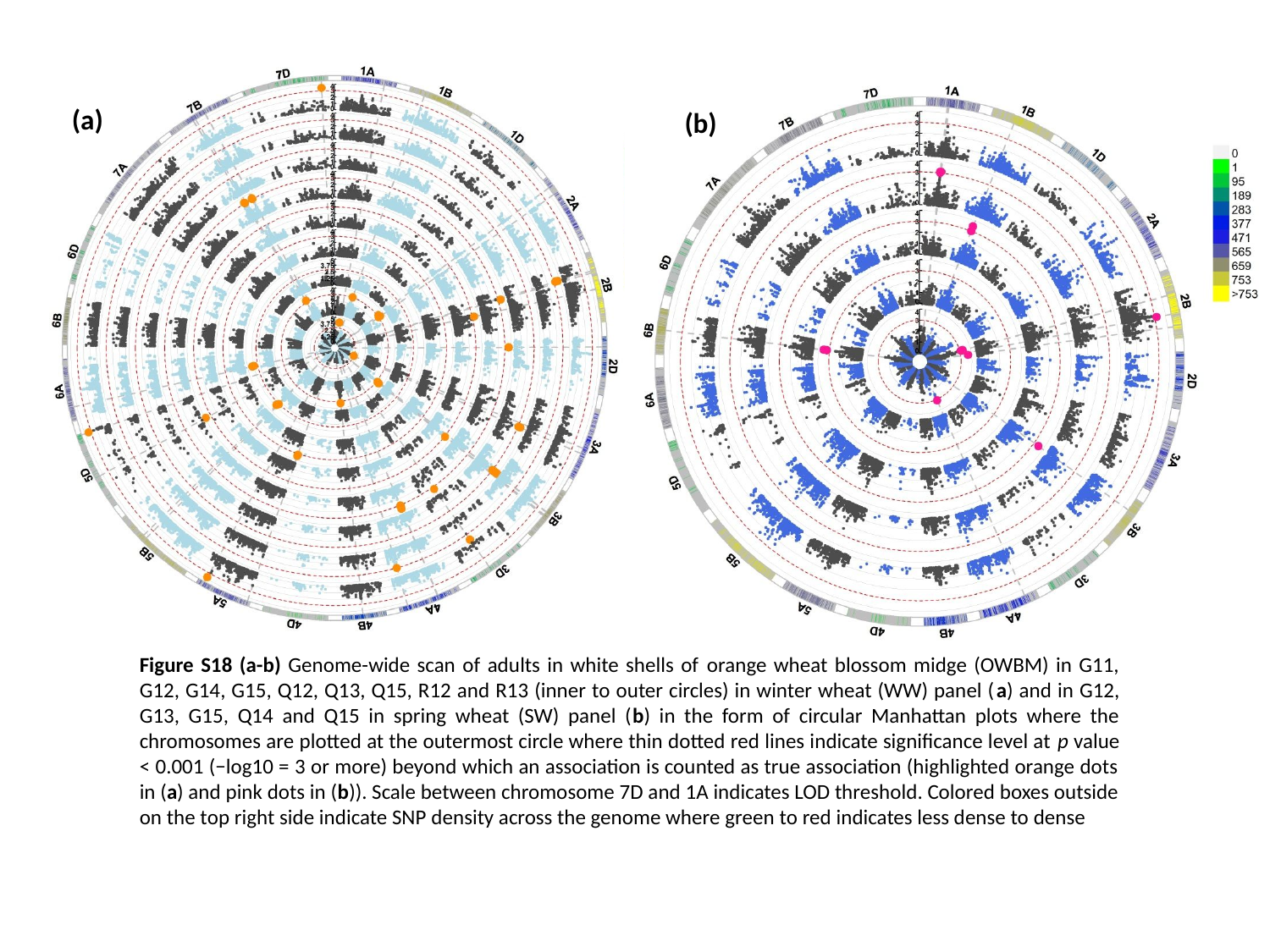

(a)
(b)
Figure S18 (a-b) Genome-wide scan of adults in white shells of orange wheat blossom midge (OWBM) in G11, G12, G14, G15, Q12, Q13, Q15, R12 and R13 (inner to outer circles) in winter wheat (WW) panel (a) and in G12, G13, G15, Q14 and Q15 in spring wheat (SW) panel (b) in the form of circular Manhattan plots where the chromosomes are plotted at the outermost circle where thin dotted red lines indicate significance level at p value < 0.001 (−log10 = 3 or more) beyond which an association is counted as true association (highlighted orange dots in (a) and pink dots in (b)). Scale between chromosome 7D and 1A indicates LOD threshold. Colored boxes outside on the top right side indicate SNP density across the genome where green to red indicates less dense to dense

## Slide 24
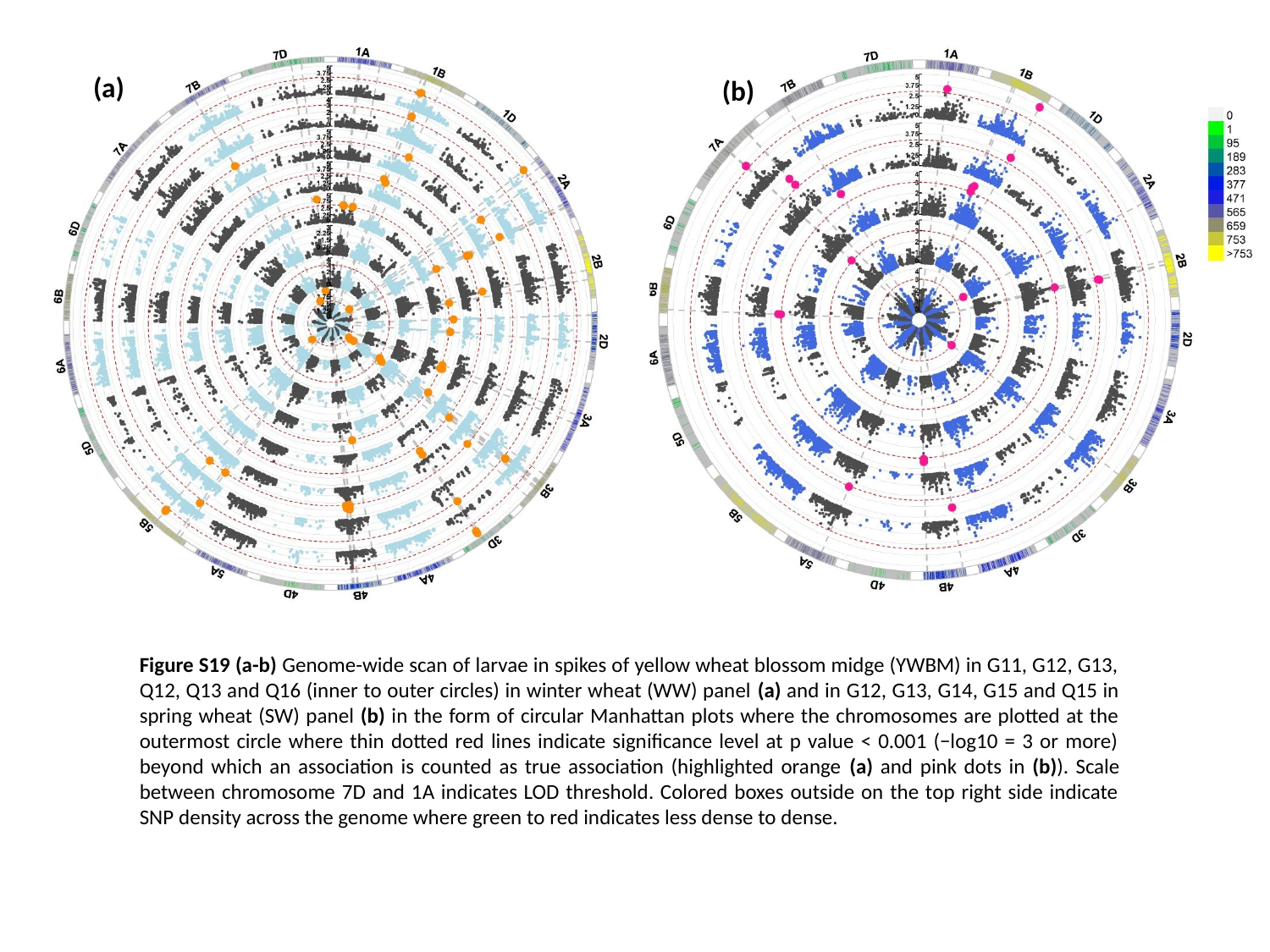

(a)
(b)
Figure S19 (a-b) Genome-wide scan of larvae in spikes of yellow wheat blossom midge (YWBM) in G11, G12, G13, Q12, Q13 and Q16 (inner to outer circles) in winter wheat (WW) panel (a) and in G12, G13, G14, G15 and Q15 in spring wheat (SW) panel (b) in the form of circular Manhattan plots where the chromosomes are plotted at the outermost circle where thin dotted red lines indicate significance level at p value < 0.001 (−log10 = 3 or more) beyond which an association is counted as true association (highlighted orange (a) and pink dots in (b)). Scale between chromosome 7D and 1A indicates LOD threshold. Colored boxes outside on the top right side indicate SNP density across the genome where green to red indicates less dense to dense.

## Slide 25
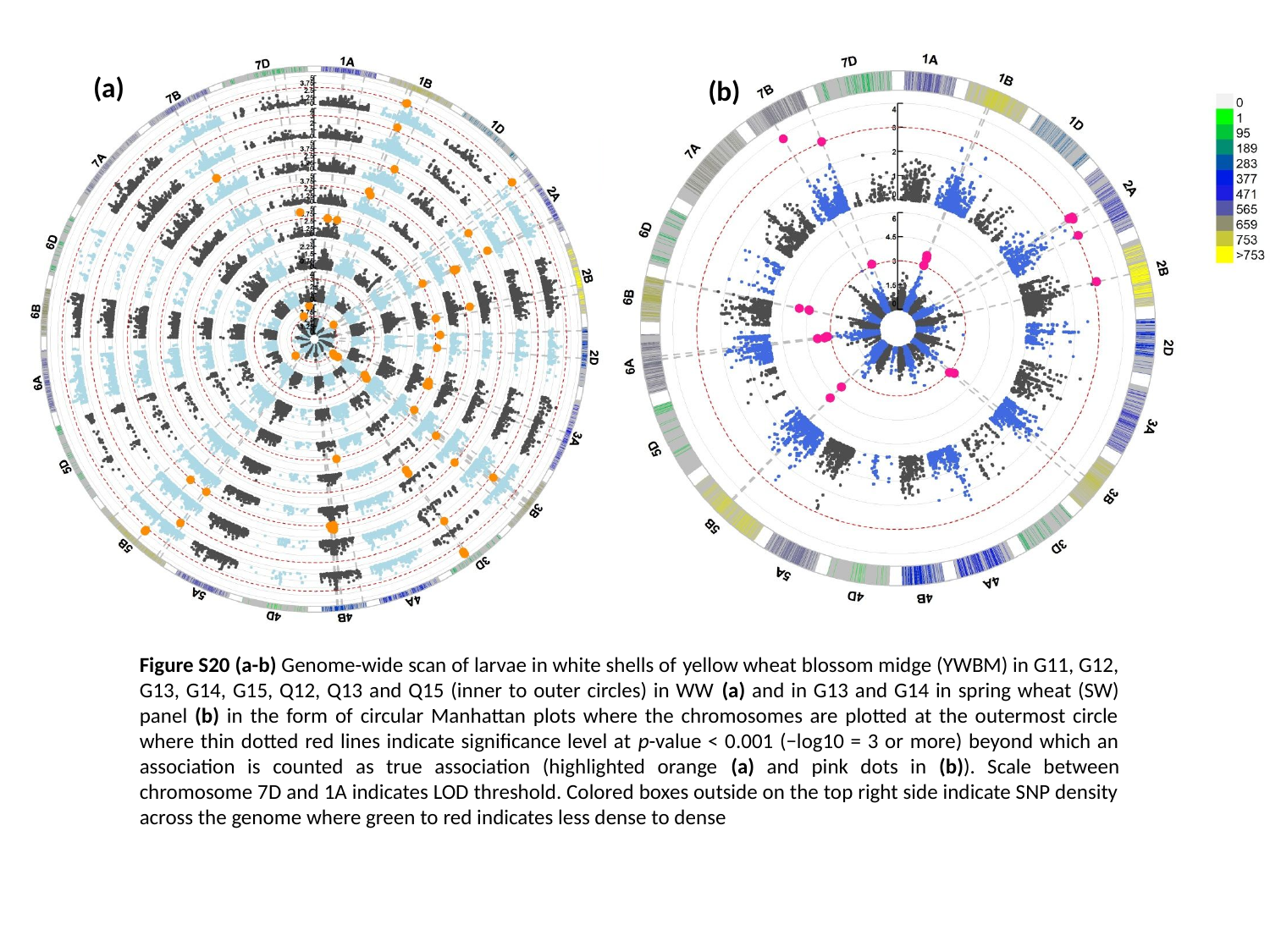

(a)
(b)
Figure S20 (a-b) Genome-wide scan of larvae in white shells of yellow wheat blossom midge (YWBM) in G11, G12, G13, G14, G15, Q12, Q13 and Q15 (inner to outer circles) in WW (a) and in G13 and G14 in spring wheat (SW) panel (b) in the form of circular Manhattan plots where the chromosomes are plotted at the outermost circle where thin dotted red lines indicate significance level at p-value < 0.001 (−log10 = 3 or more) beyond which an association is counted as true association (highlighted orange (a) and pink dots in (b)). Scale between chromosome 7D and 1A indicates LOD threshold. Colored boxes outside on the top right side indicate SNP density across the genome where green to red indicates less dense to dense

## Slide 26
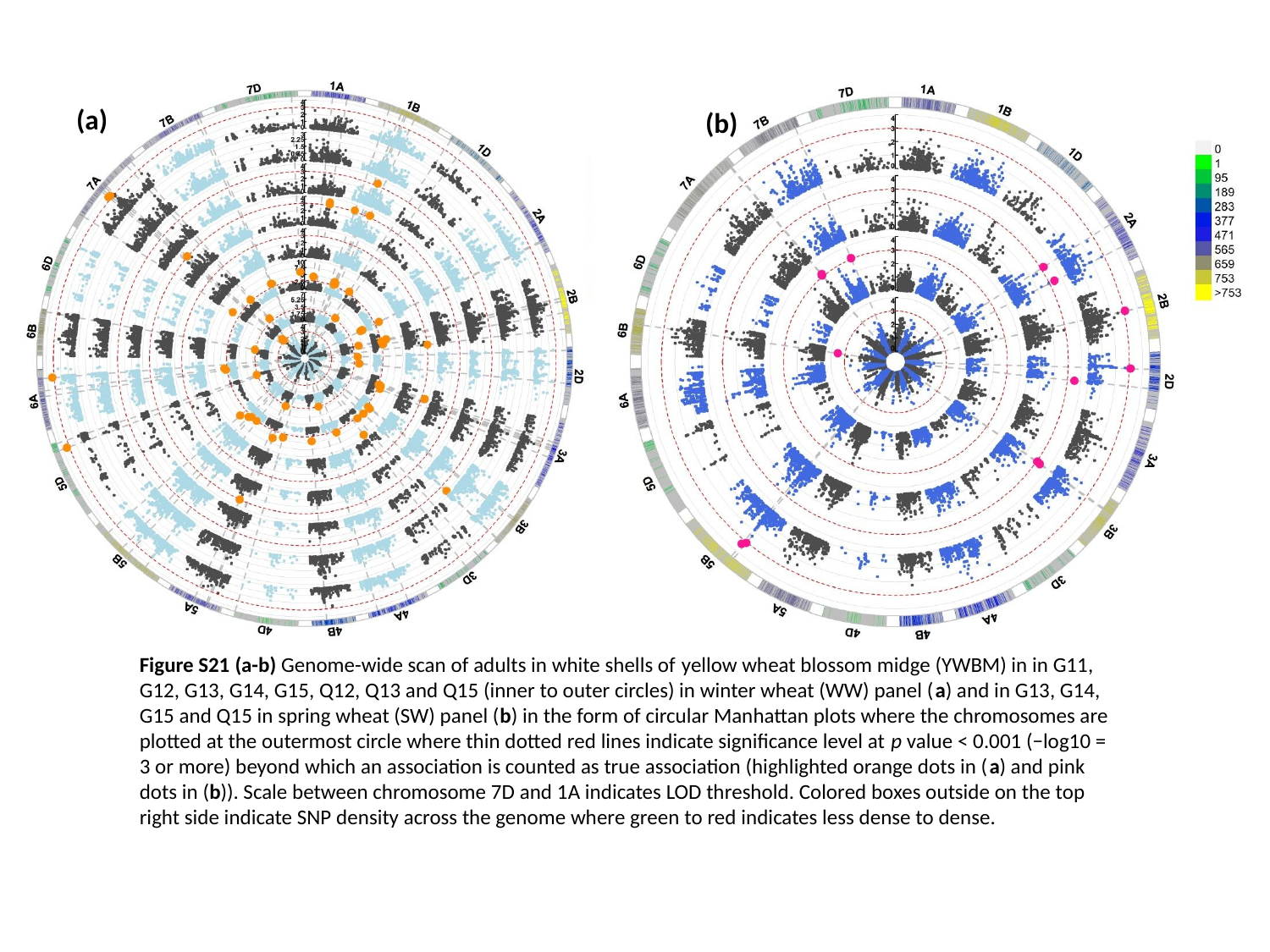

(a)
(b)
Figure S21 (a-b) Genome-wide scan of adults in white shells of yellow wheat blossom midge (YWBM) in in G11, G12, G13, G14, G15, Q12, Q13 and Q15 (inner to outer circles) in winter wheat (WW) panel (a) and in G13, G14, G15 and Q15 in spring wheat (SW) panel (b) in the form of circular Manhattan plots where the chromosomes are plotted at the outermost circle where thin dotted red lines indicate significance level at p value < 0.001 (−log10 = 3 or more) beyond which an association is counted as true association (highlighted orange dots in (a) and pink dots in (b)). Scale between chromosome 7D and 1A indicates LOD threshold. Colored boxes outside on the top right side indicate SNP density across the genome where green to red indicates less dense to dense.

## Slide 27
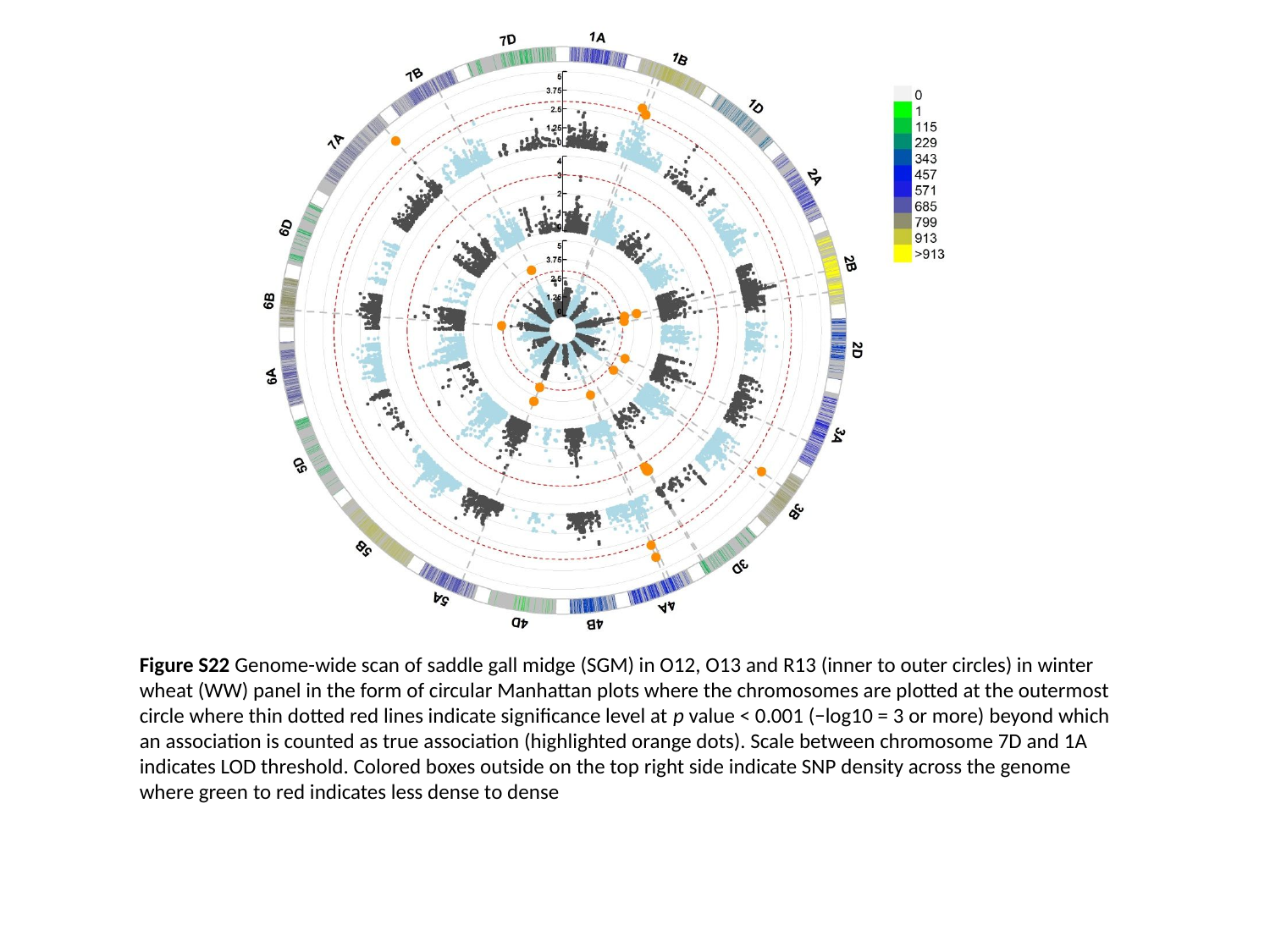

Figure S22 Genome-wide scan of saddle gall midge (SGM) in O12, O13 and R13 (inner to outer circles) in winter wheat (WW) panel in the form of circular Manhattan plots where the chromosomes are plotted at the outermost circle where thin dotted red lines indicate significance level at p value < 0.001 (−log10 = 3 or more) beyond which an association is counted as true association (highlighted orange dots). Scale between chromosome 7D and 1A indicates LOD threshold. Colored boxes outside on the top right side indicate SNP density across the genome where green to red indicates less dense to dense

## Slide 28
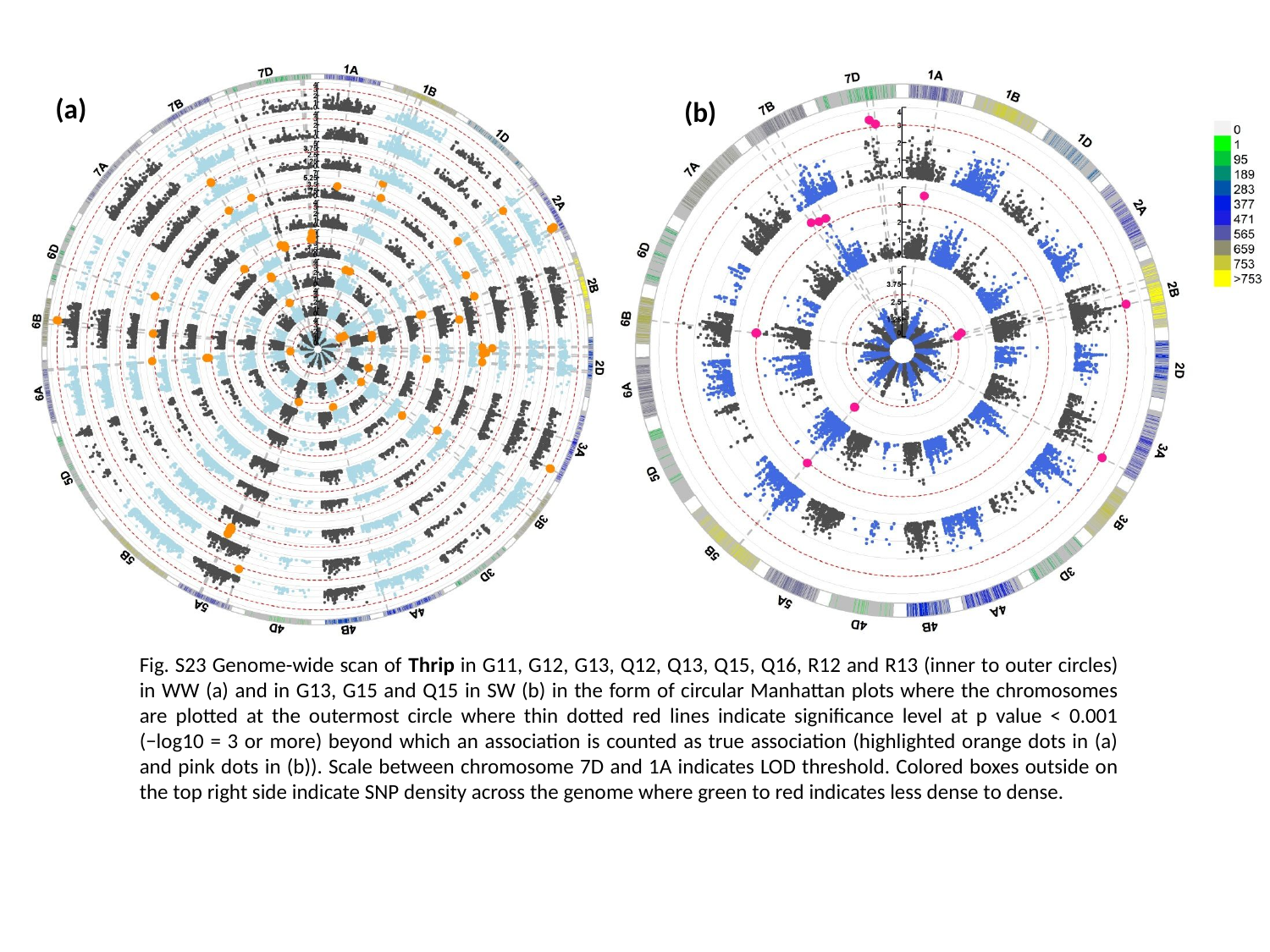

(a)
(b)
Fig. S23 Genome-wide scan of Thrip in G11, G12, G13, Q12, Q13, Q15, Q16, R12 and R13 (inner to outer circles) in WW (a) and in G13, G15 and Q15 in SW (b) in the form of circular Manhattan plots where the chromosomes are plotted at the outermost circle where thin dotted red lines indicate significance level at p value < 0.001 (−log10 = 3 or more) beyond which an association is counted as true association (highlighted orange dots in (a) and pink dots in (b)). Scale between chromosome 7D and 1A indicates LOD threshold. Colored boxes outside on the top right side indicate SNP density across the genome where green to red indicates less dense to dense.

## Slide 29
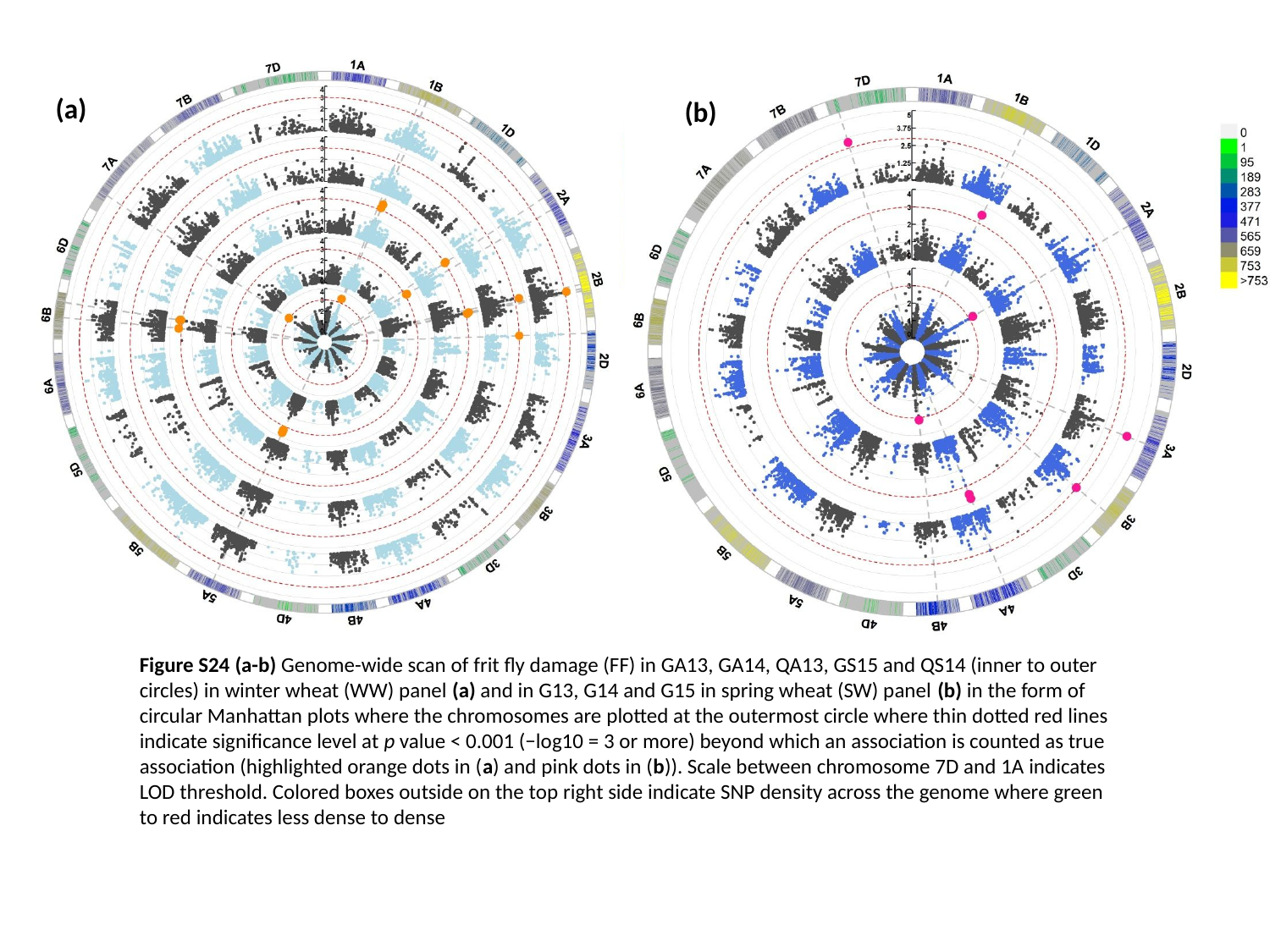

(a)
(b)
Figure S24 (a-b) Genome-wide scan of frit fly damage (FF) in GA13, GA14, QA13, GS15 and QS14 (inner to outer circles) in winter wheat (WW) panel (a) and in G13, G14 and G15 in spring wheat (SW) panel (b) in the form of circular Manhattan plots where the chromosomes are plotted at the outermost circle where thin dotted red lines indicate significance level at p value < 0.001 (−log10 = 3 or more) beyond which an association is counted as true association (highlighted orange dots in (a) and pink dots in (b)). Scale between chromosome 7D and 1A indicates LOD threshold. Colored boxes outside on the top right side indicate SNP density across the genome where green to red indicates less dense to dense
